# Formation of alkyne-bridged ferrocenophanes using ring-closing alkyne metathesis on 1,1’-diacetylenic ferrocenes

**DOI:** 10.3762/bjoc.15.246

**Published:** 2019-10-24

**Authors:** Celine Bittner, Dirk Bockfeld, Matthias Tamm

**Affiliations:** 1 Institut für Anorganische und Analytische Chemie, Technische Universität Braunschweig, Hagenring 30, 38102 Braunschweig, Germany

**Keywords:** alkyne metathesis, ferrocene, homogeneous catalysis, molybdenum, terminal alkynes

## Abstract

Novel alkyne-bridged ferrocenophanes [fc{CO_2_(CH_2_)*_n_*C≡}_2_] (**2a**: *n* = 2; **2b**: *n* = 3) were synthesized from the corresponding terminal diacetylenic ferrocenes [fc{CO_2_(CH_2_)*_n_*C≡CH}_2_] (**1a**: *n* = 2; **1b**: *n* = 3) through ring-closing alkyne metathesis (RCAM) utilizing the highly effective molybdenum catalyst [MesC≡Mo{OC(CF_3_)_2_CH_3_}_3_] (**MoF6**; Mes = 2,4,6-trimethylphenyl). The metathesis reaction occurs in short time with high yields whilst giving full conversion of the terminal alkynes. Furthermore, the solvent-dependant reactivity of **2a** towards Ag(SbF_6_) is investigated, leading to oxidation and formation of the ferrocenium hexafluoroantimonate **4** in dichloromethane, whereas the silver(I) coordination polymer **5** was isolated from THF solution.

## Introduction

**Figure 1:**
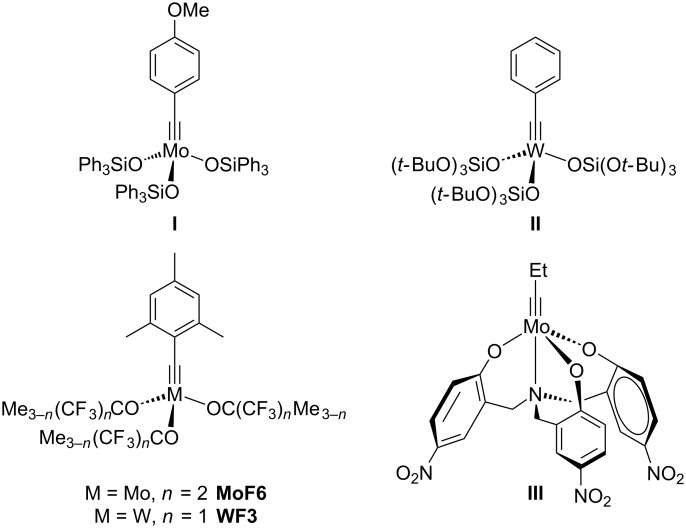
Well-defined catalysts for alkyne metathesis.

Alkyne metathesis, the reversible making and breaking of carbon–carbon triple bonds, is clearly gaining more attention. Not only could a great number of active catalysts for alkyne metathesis be developed over the past decades, but also their field of applications is steadily growing [1–7]. Numerous symmetric complexes of the Schrock type [RC≡MX_3_] [8–9] bearing a great variety of ancillary ligands X were successfully explored for several types of alkyne metatheses. A selection of such complexes is shown in Figure 1. Molybdenum alkylidyne complex **I** with siloxide ligands (X = OSiPh_3_) [10–11] is widely used in natural product synthesis, predominantly through ring-closing alkyne metathesis (RCAM) [12–20].

The metathesis of conjugated diynes (DYCM) is promoted by the benzylidyne tungsten complex **II** with silanolate ligands (X = OSi(O*t-*Bu)_3_) [21–22]; this catalyst is also active in classical alkyne metathesis [23]. Complex **III** with chelating phenoxide ligands [24–27] is mostly applied in supramolecular chemistry [28–32], used for instance in the preparation of aryleneethynylene macrocycles and cages through alkyne metathesis [33–39]. Additionally, already since the 1980s the influence of fluorinated and unfluorinated alkoxide ligands has been widely investigated [40–41]. Only recently, we were able to present a molybdenum complex decorated with hexafluoro-*tert*-butoxide ligands [MesC≡Mo{OC(CH_3_)(CF_3_)_2_}_3_] (**MoF6**; Mes = 2,4,6-trimethylphenyl) which proved to be highly active in the metathesis of internal and even terminal alkynes [42–44]. The same accounts for the recently added tungsten complex [MesC≡W{OC(CH_3_)_2_(CF_3_)}_3_] (**WF3**) [45]. Because of the higher electrophilicity of the tungsten metal, the less electron-rich trifluoro-*tert*-butoxide ligands are sufficient to obtain an active catalyst for the metathesis of terminal alkynes. This reactivity could only lately be shown for complex **I** as well [13,16,46]. Beforehand, the promotion of terminal alkyne metathesis (TAM) proved to be difficult due to several deactivation pathways [47–53].

Regarding the metathesis of organometallic substrates, numerous examples of a conversion via olefin metathesis can be found in the literature [54–57], including the formation of olefinic metallocenophanes via ring-closing olefin metathesis [58–61], and the preparation of supramolecular structures using template synthesis [62–69]. However, only few cases are illustrated for alkyne metathesis reactions of organometallic compounds. After some stoichiometric reactions of ruthenium and rhenium half sandwich complexes [70], several reactions have been described exploiting the reactivity of acetylenic ferrocene compounds [71–75]. For most of these conversions the Mortreux catalyst system Mo(CO)_6_/ArOH was used at elevated temperatures. Because of the electronic properties of the ferrocene unit, polymers of acetylenic ferrocenes can be used for example as molecular wires [75].

**Figure 2:**
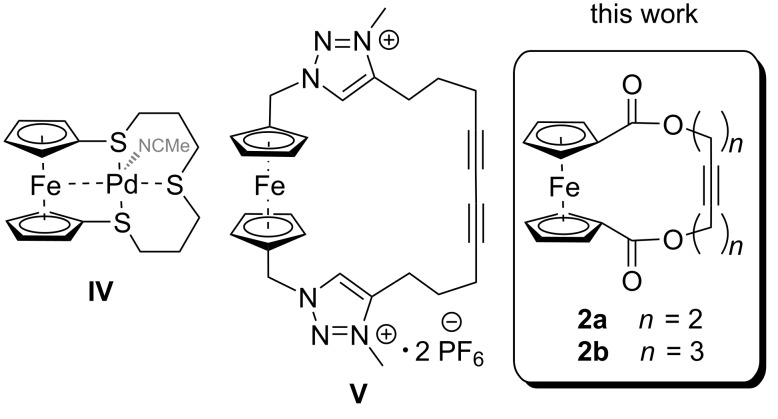
Examples for a ferrrocenic thiacrown ether complexing palladium (**IV**), and a dicationic ferrocenophane (**V**) featuring a diyne bridge for ion sensing.

Coming to ferrocenophanes, mainly carbon-bridged compounds are known [76–77]. Additionally, some complexes of mostly soft transition metals with ferrocenophane ligands could be identified. Herein, a main focus lies on complexes with ferrocenic thiacrown ethers as well as selenacrown ethers coordinating a transition metal occasionally inside the cavity of the ferrocenophane [78–81]. In some cases, a metal–iron interaction, e.g., as shown in complex **IV** [79], could be observed depending on the ring size of the ferrocenophane ligand [82–85]. Furthermore, ferrocenophanes functionalized with bridges containing various heteroatoms were utilized as ion sensors for a wide variety of applications [86–90]. An example (complex **V**) with an additional diyne moiety is given in Figure 2 [91].

To the best of our knowledge, no ferrocenophanes formed via RCAM have been reported to date. With this contribution, we would like to present the first RCAM of 1,1’-diacetylenic ferrocenes **1** (**a**: *n* =2; **b**: *n* = 3) to form the corresponding alkyne-bridged ferrocenophanes **2** (**a**: *n* =2; **b**: *n* = 3; Figure 2). Besides these ferrocenophanes being the first to be formed in an alkyne metathesis reaction, they are additionally derived from terminal alkynes using the complex **MoF6** as a catalyst. Furthermore, the ability of the new [10]ferrocenophane **2a** to bind transition metal cations is outlined, and the redox properties of the new ferrocenophanes are studied by cyclovoltammetry.

## Results and Discussion

**Scheme 1:**
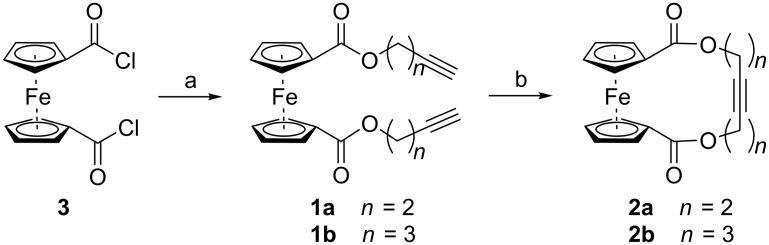
Synthesis of substrates **1** (**a** *n* = 2; **b** *n* = 3) via esterification of **3** and following RCAM with catalyst **MoF6** to ferrocenophanes **2** (**a**: *n* = 2; **b**: *n* = 3); a) HO(CH_2_)*_n_*C≡CH (*n =* 2, 3), DMAP, NEt_3_, DCM, 0 °C to rt; b) 2 mol % **MoF6**, MS 5Å, toluene, rt.

The substrates **1a** and **1b** for the RCAM toward the desired ferrocenophanes **2a** and **2b** were synthesized via an esterification reaction starting from symmetrical 1,1’-ferrocenoyl dichloride (**3**) [92] as shown in Scheme 1. To a solution of the corresponding 1-alkynol (13.5 mmol), NEt_3_ (13.5 mmol), and 4-dimethylaminopyridine (DMAP, 0.5 mmol) in dichloromethane (DCM, 20 mL) at 0 °C the dichloride **3** (2 g, 6.4 mmol) in DCM (20 mL) was added slowly via a dropping funnel. The resulting dark orange solution was stirred overnight at room temperature to yield the desired symmetrical 1,1’-ferrocene diacetylenes **1a** (*n* = 2) and **1b** (*n* = 3) in high yields of 82% and 94%, respectively. The compounds were obtained as orange powders after aqueous work-up and column chromatography on silica gel.

**Figure 3:**
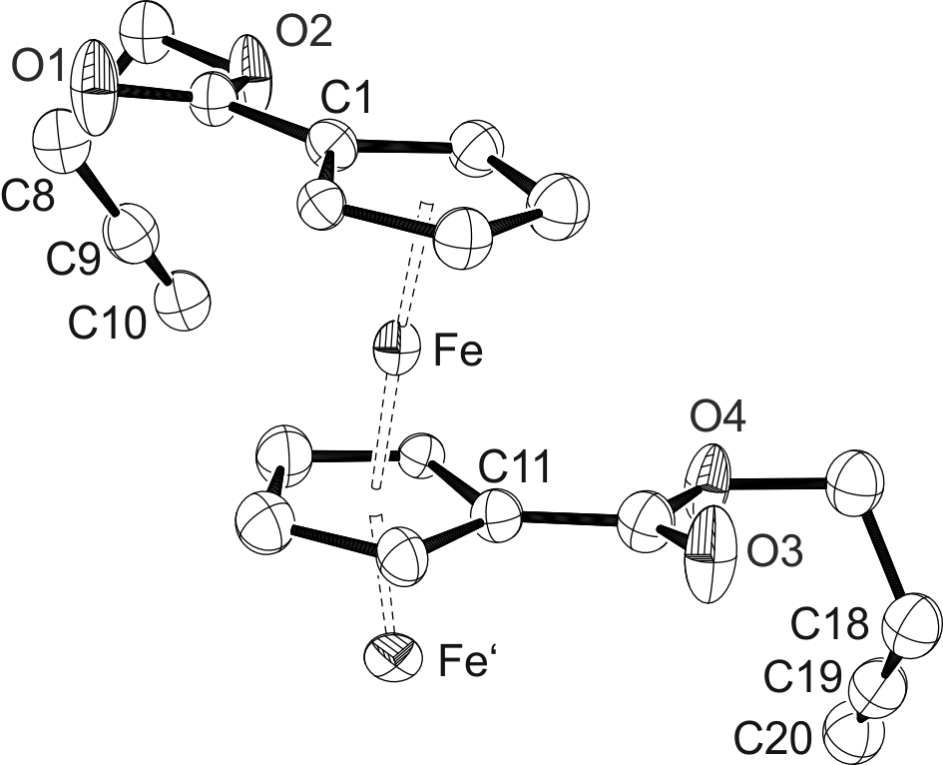
ORTEP diagram of **1a** with thermal displacement parameters drawn at 50% probability; hydrogen atoms are omitted for clarity; occupation Fe 74%, Fe’ 26%. Selected bond lengths [Å] and angles [°]: Fe–Ct1 1.6947(12), Fe-Ct11 1.6695(12), Ct1-Fe-Ct11 176.96(8), C9–C10 1.186(11), C8–C9–C10 177.2(9), C19–C20 1.165(12), C18–C19–C20 178.1(10).

**Figure 4:**
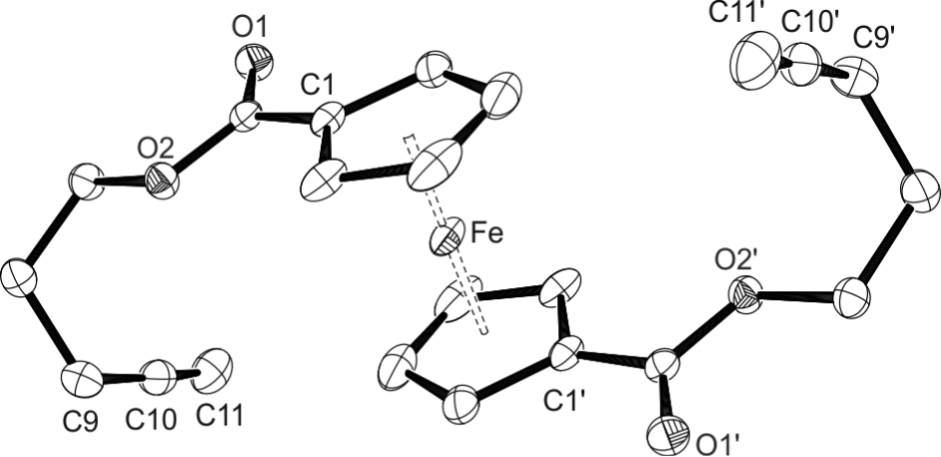
ORTEP diagram of **1b** with thermal displacement parameters drawn at 50% probability; hydrogen atoms are omitted for clarity. Selected bond lengths [Å] and angles [°]: Fe–Ct 1.6523(7), Ct–Fe–Ct‘ 180.0, C10–C11 1.127(2), C9–C10–C11 176.3(2).

The substrates show the characteristic signals for the AA’BB’ line system of the ferrocene protons in the ^1^H NMR spectrum at chemical shifts of 4.85 ppm and 4.43 ppm for **1a**, and at 4.83 ppm and 4.42 ppm for **1b**. The acetylenic sidechains show the characteristic triplet with a small coupling constant for the terminal alkyne proton at chemical shifts of 2.05 ppm and 2.01 ppm, respectively. The NMR data for the butynyl compound **1a** fit the results of Suitor et al. that were published only recently [93]. From saturated DCM solutions of **1a** and **1b** after layering with pentane or hexane, respectively, crystals suitable for X-ray diffraction analysis could be obtained. The corresponding ORTEP drawings are given in Figure 3 and Figure 4. Ferrocene **1a** crystallises in the orthorhombic space group *Pca*2_1_, while the analogues compound **1b** crystallises in the monoclinic space group *P*2_1_/*c*. The asymmetric unit for the molecular structure of **1b** shows only half a molecule with the other half being generated through an inversion centre located on the iron atom. For both structures, the iron-centroid (Fe–Ct) distances of 1.6947(12) Å (Fe–Ct1) and 1.6695(12) Å (Fe–Ct11) in **1a** as well as of 1.6523(7) Å (Fe–Ct) in **1b** are in the range of other 1,1’-substituted ferrocenes. The same can be observed for the C≡C triple bonds with bond lengths of 1.186(11) Å and 1.165(12) Å in **1a** and 1.127(2) Å in **1b**, which are only slightly shorter than in ethyne [94]. Interestingly, a disorder of the iron position in the butynylferrocene **1a** can be observed, obviously being responsible for the slightly longer Fe–Ct distances as compared to **1b**. The main position Fe has an occupation of 74%. The disordered position Fe’ with an occupation of 26% is located between the centroid of C11 and the centroid of C1’ of the following ferrocene unit connected through the main position Fe resulting in a chain like structure of ferrocenes. An ORTEP drawing of this structure can be found in the Supporting Information File 1 (Figure S15). Because of this disorder the cyclopentadienyl rings should disclose alternate positions as well, however, the central position of Fe’ prevents such observation. Nevertheless, it should be taken into account that the discussed structural parameters for **1a** should not be viewed representative for the molecular structure of **1a** as a result of the disorder on the iron position. Unfortunately, no single crystals for X-ray diffraction analysis could be obtained with such disorder being absent.

**Figure 5:**
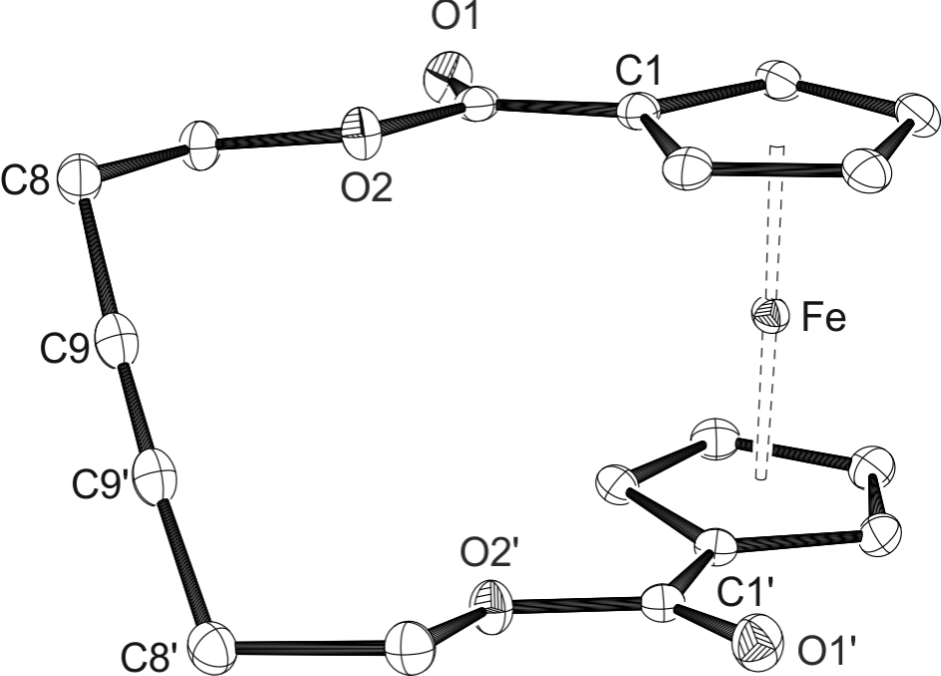
ORTEP diagram of **2a** with thermal displacement parameters drawn at 50% probability; hydrogen atoms are omitted for clarity. Selected bond lengths [Å] and angles [°]: Fe–Ct 1.6414(6), Ct–Fe–Ct’ 178.809(1), C9–C9‘ 1.193(2), C8–C9–C9‘ 172.1(2).

For the catalytic RCAM (Scheme 1), the substrates **1a** and **1b** were dissolved in toluene at high dilution (4.5 mM), and the catalyst **MoF6** (2 mol %) was added as a solid. The metathesis reactions were performed in the presence of molecular sieves with a pore size of 5 Å (MS 5 Å) to absorb acetylene which is formed during the reaction. The products of the metathesis reactions could be obtained as orange solids. The RCAM of the butynyl system **1a** afforded the desired monomeric ferrocenophane **2a** in a high yield (93%). The signal for the terminal proton has vanished in the ^1^H NMR spectrum, and the ^13^C NMR spectrum only shows one signal at 79.6 ppm associated with the symmetric C≡C triple bond. The same high yield for compound **2a** could be achieved in an identically performed RCAM with a higher substrate concentration of **1a** of 21 mM. When further increasing the concentration of **1a** to 125 mM a product mixture of the monomeric ferrocenophane **2a**, the fully ring-closed dimeric compound as well as the open dimer can be identified with the help of mass spectrometry (see also experimental section in Supporting Information File 1). Crystals of **2a** suitable for X-ray diffraction analysis could be obtained from a hot saturated solution in toluene after cooling to −28 °C. An ORTEP drawing is given in Figure 5.

The ferrocenophane **2a** crystallises in the orthorhombic space group *Pbcn* with half a molecule in the asymmetric unit. The whole molecule is generated via a 2-fold axis. In comparison with the dialkynyl substrate **1a**, the distances of 1.6414(6) Å from the iron atom to the centroids is slightly shorter with a Ct–Fe–Ct’ angle of 178.809(1)° being marginally closer to linearity. In contrast, the angle of the C≡C triple bond with a value of 172.1(2)° is smaller as a result of the now occurring ring tension. A polymorph of the structure of **2a** crystallising in the triclinic space group 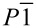 could be obtained after crystallisation with Et_2_O from a saturated solution of **2a** in THF. An ORTEP diagram of the polymorphous structure is displayed in Figure S16 in Supporting Information File 1.

**Figure 6:**
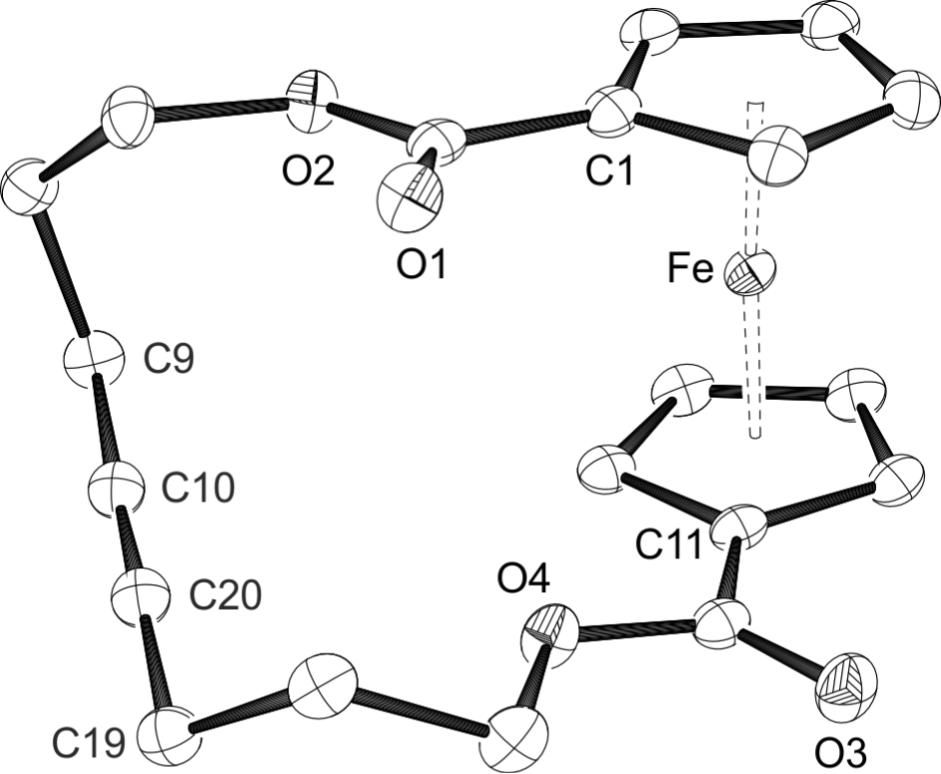
ORTEP diagram of **2b** (one of two molecules of the asymmetric unit) with thermal displacement parameters drawn at 50% probability; hydrogen atoms are omitted for clarity. Selected bond lengths [Å] and angles [°]: Fe–Ct1 1.6558(2), Fe–Ct11 1.6548(2), Ct1–Fe–Ct11 176.83(2), C10–C20 1.198(3), C9–C10–C20 177.6(2), C10–C20–C19 179.1(2).

As for the pentynyl substrate **1b**, already with a high dilution of the reaction mixture of 4.5 mM a mixture of two ferrocene-containing compounds can be observed in the ^1^H NMR spectrum. Certainly, complete conversion of **1b** could be achieved in the metathesis reaction after 4 hours as the signal for the terminal proton cannot be observed anymore in the ^1^H NMR spectrum. With the aid of mass spectrometry, the two species could be assigned as the monomeric ferrocenophane **2b** as well as its fully ring-closed dimeric analogue of the formula [Fe{C_5_H_4_COO(CH_2_)_3_ C≡}_2_]_2_ in a ratio of approximately 4:1. Related NMR spectra can be found in Supporting Information File 1 (Figures S9 and S10). Separation of the monomer **2b** could be achieved with column chromatography on silica gel with a yield of 53%. The ring-closed dimer remained in a mixture with excess monomer. Single crystals of compound **2b** suitable for X-ray diffraction analysis could be obtained at room temperature from a DCM or CDCl_3_ solution layered with hexane, respectively. An ORTEP drawing of **2b** is shown in Figure 6. The ferrocenophane crystallises in the triclinic space group 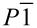 with two molecules in the asymmetric unit. For convenience, only one molecule is displayed in Figure 6, an ORTEP diagram of both molecules can be found in Figure S17 in Supporting Information File 1. In contrast to the [10]ferrocenophane **2a** the carbonyl groups in **2b** are facing the same direction and as a result of the larger ring size the alkyne moiety is laying almost in the plane of Ct11. The dimeric compound unfortunately could not be crystallised from the mixture with **2b**.

Interestingly, in the case of the butynyl substrate **1a** exclusively the monomeric ring closure to ferrocenophane **2a** occurs using standard metathesis conditions that remains even upon establishing a higher substrate concentration. Therefore, the monomeric ferrocenophane **2a** needs to be energetically considerably more stable than its dimeric congener. In contrast, the pentynyl substrate **1b** already gives mixtures of monomeric and dimeric ring-closed products at high dilution (4.5 mM), which means the energy gap between these compounds has become significantly smaller. The equilibria that are established within a metathesis reaction depend on the equilibrium rate constants, in this case between the monomeric and dimeric ring-closed products. As shown before by our group with other cyclophanes, these can also be determined theoretically to predict the synthetic outcome of a metathesis reaction [95]. To further complete the series of diacetylenic ferrocene substrates for the catalytic RCAM the analogous propargylic compound was synthesized as well in high yield. The NMR data fit the results of Suitor et al. that were published only recently [93]. Unfortunately, the propargylic compound could not be characterised crystallographically as it only gave an orange powder upon different crystallisation techniques. In the following catalytic conversion, no ring closure could be observed as the proton NMR spectrum showed the starting material exclusively with the characteristic signals for the terminal proton of the C≡CH triple bond at 2.52 ppm. However, this behaviour is in good agreement with other literature examples that state the metathesis of internal propargylic systems being particularly challenging [13,96–97].

**Figure 7:**
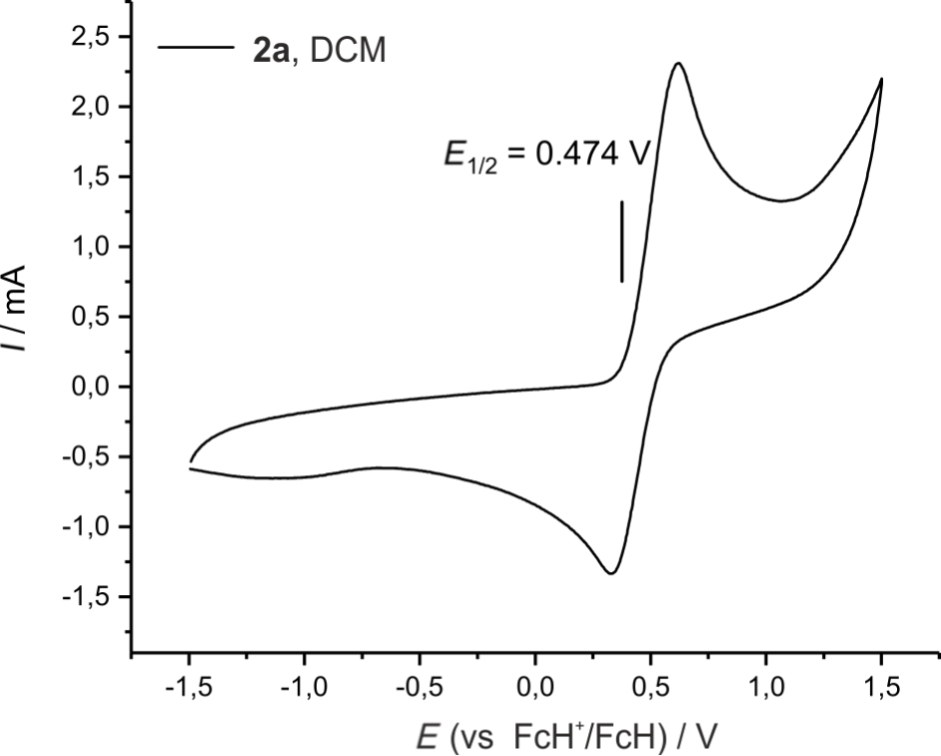
Cyclic voltammogram of **2a** in DCM, 0.2 M *n-*Bu_4_NPF_6_, 1 V s^−1^ scan rate, referenced vs FcH/FcH ^+^.

As the [10]ferrocenophane **2a** can be obtained reliably and selectively from the RCAM reaction, additional electrochemical and chemical studies were performed on this compound. To resolve the barrier for the chemical oxidation, the cyclovoltammogram of **2a** was recorded in DCM (Figure 7). The quasi-reversible redox process assigned to the Fe(II)/Fe(III) couple occurs at a potential of *E*_1/2_ = 0.474 V relative to FcH/FcH ^+^. The electron-withdrawing features of the acetylenic diester bridge result in the higher potential compared to the standard system FcH/FcH ^+^ showing that the ferrocenophane **2a** is harder to oxidize than pure ferrocene. As expected, no further oxidation or reduction step could be detected.

**Scheme 2:**
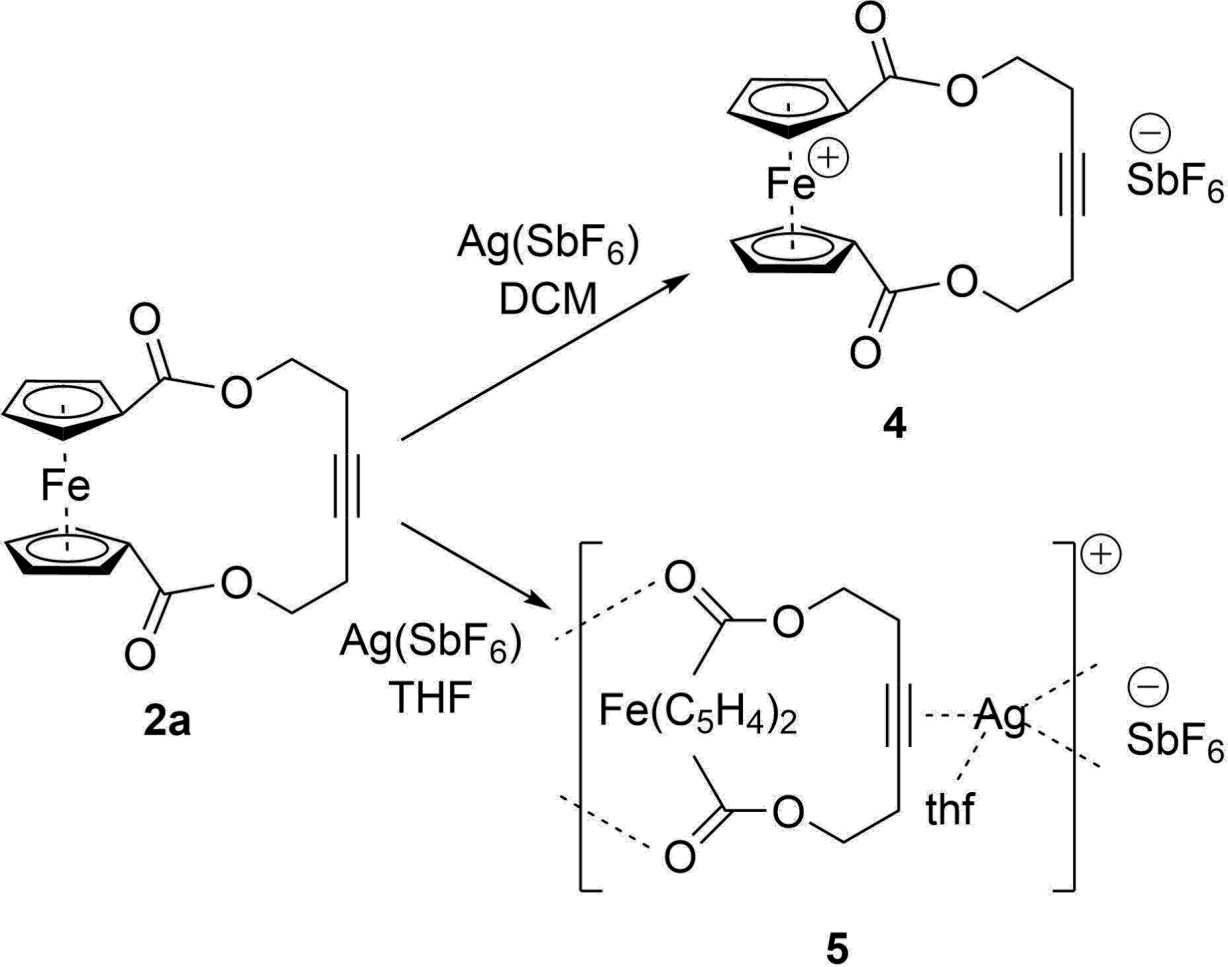
Top: Oxidation of ferrocenophane **2a** to the corresponding ferrocenium cation **4** with Ag(SbF_6_) in DCM solution; bottom: upon reaction of **2a** with Ag(SbF_6_) in THF the formation of coordination polymer **5** is observed.

**Figure 8:**
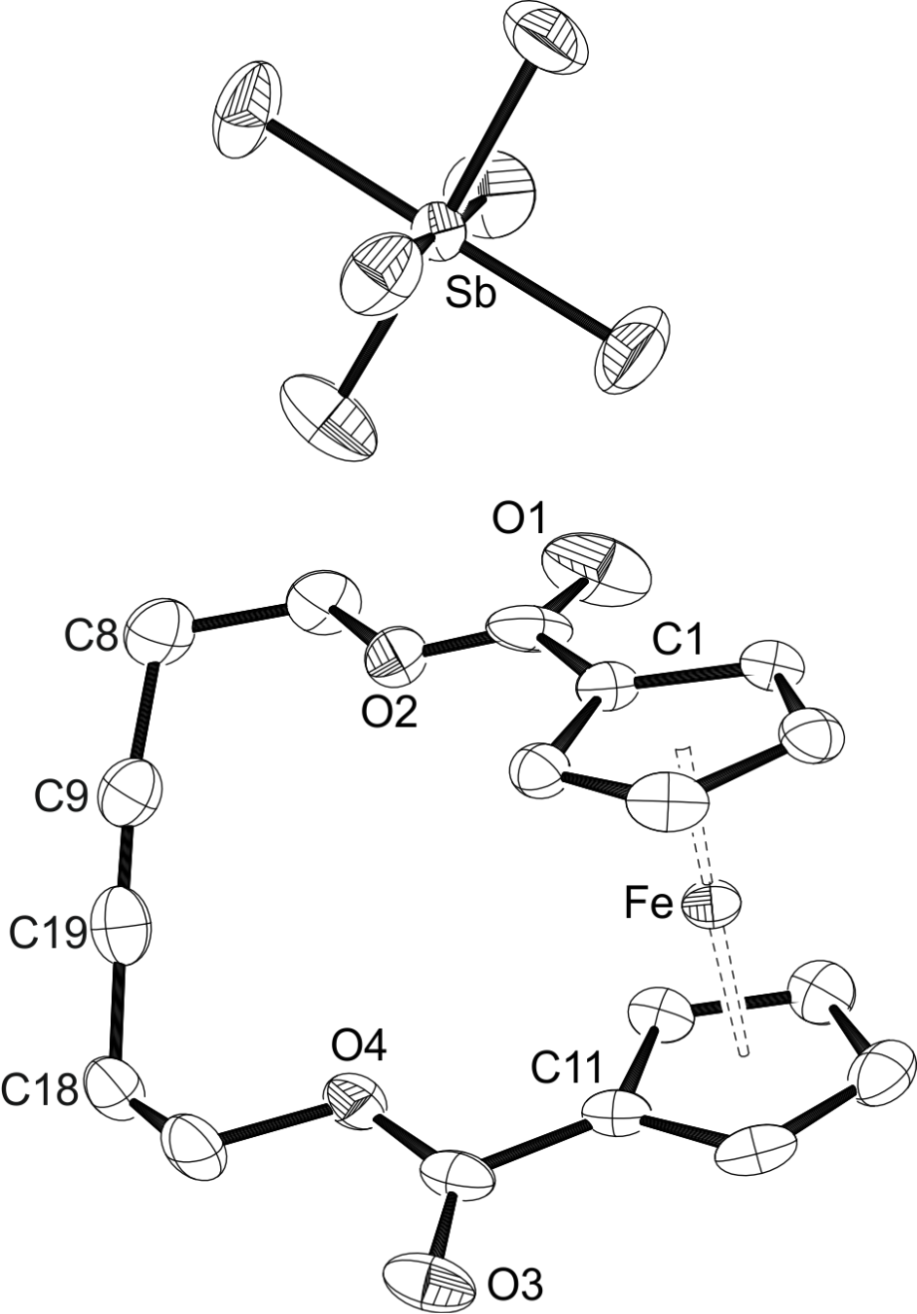
ORTEP diagram of **4** with thermal displacement drawn at 50% probability; hydrogen atoms are omitted for clarity. Disordered positions from O2 to C19, only the main component is shown. Selected bond lengths [Å] and angles [°]: Fe–Ct1 1.7100(4), Fe–Ct11 1.7131(4), Ct1–Fe–Ct11 178.11(3).

The redox potential of 0.474 V established for **2a** in DCM falls in the range of formal potentials recorded for the Ag^+^/Ag couple, which are strongly solvent dependent and vary from 0.04 V in acetonitrile to 0.65 V in DCM [98]. Therefore, oxidation should occur in the latter solvent, while silver(I) complexation could be expected in more coordinating solvents. Consequently, addition of a solution of Ag(SbF_6_) (1 equiv) in DCM to a solution of **2a** in DCM resulted in an immediate colour change from orange to dark green alongside with precipitation of a black solid, indicating the formation of elemental silver. A dark solid was isolated after filtration and evaporation of the solvent. Crystallisation from a saturated solution of DCM layered with hexanes finally yielded 96% of the ferrocenium compound **4** as blue needles, which were suitable for X-ray diffraction analysis (Scheme 2). An ORTEP diagram of the molecular structure of **4** can be seen in Figure 8. The oxidized ferrocenophane **4** crystallises in the monoclinic space group *P*2_1_/*c* with the carbon chain showing a disorder over five positions including the atoms O2 to C19. Compared to the neutral compound **2a** slightly longer Fe–Ct distances can be observed as expected for the removal of one electron at the central iron atom. The geometry around the iron centre is viewed linear with an angle of 178.11(3)°. The paramagnetic nature of the oxidized ferrocenophane **4** can also be verified using ^1^H NMR analysis. While the signals for the CH_2_ groups of the carbon bridge occur as a multiplet at 1.37–1.29 ppm as well as a triplet at 0.90 ppm, the signals for the ferrocene protons can be found in the paramagnetic area as broad singlets with chemical shifts of −3.80 ppm and −5.43 ppm.

**Figure 9:**
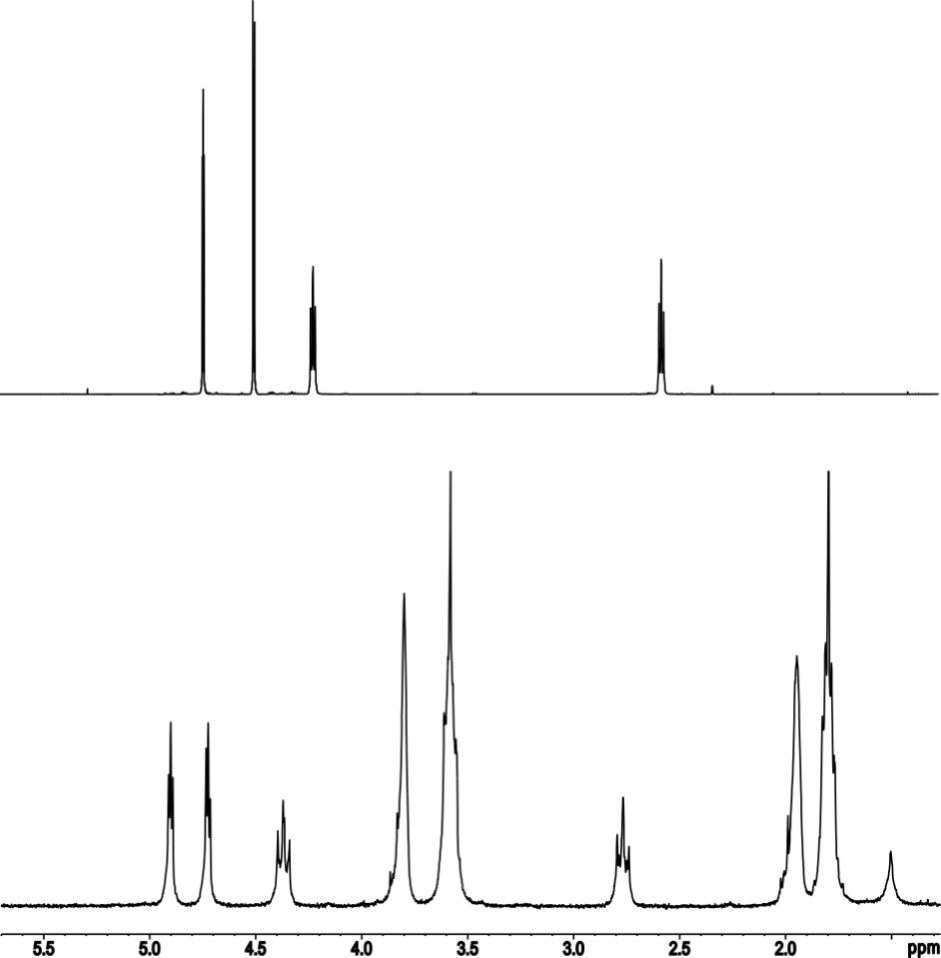
^1^H NMR (200.1 MHz, 298 K) spectrum of top: **2a** in CDCl_3_; bottom: **5** in THF-*d*_8_ – signals for solvate THF occur at 3.80 ppm and 1.95 ppm.

When the reaction of **2a** with Ag(SbF_6_) is performed in THF as a solvent, the oxidation to the ferrocenium hexafluoroantimonate **4** is not observed (Scheme 2, bottom). Upon the addition of a solution of **2a** in THF to a solution of Ag(SbF_6_) (1 equiv) in THF, the colour of the reaction mixture stayed orange for the whole reaction time. After 16 hours at room temperature, the resulting orange suspension was filtered, and the solvent was evaporated. The crude product was crystallised with hexane from a saturated solution of THF to yield compound **5** as orange crystals with a yield of 78%. The ^1^H NMR spectrum confirmed one molecule coordinating THF. All proton signals of the ferrocenophane protons are broadened and slightly shifted to lower field upon coordination of silver as can be seen in Figure 9.

**Figure 10:**
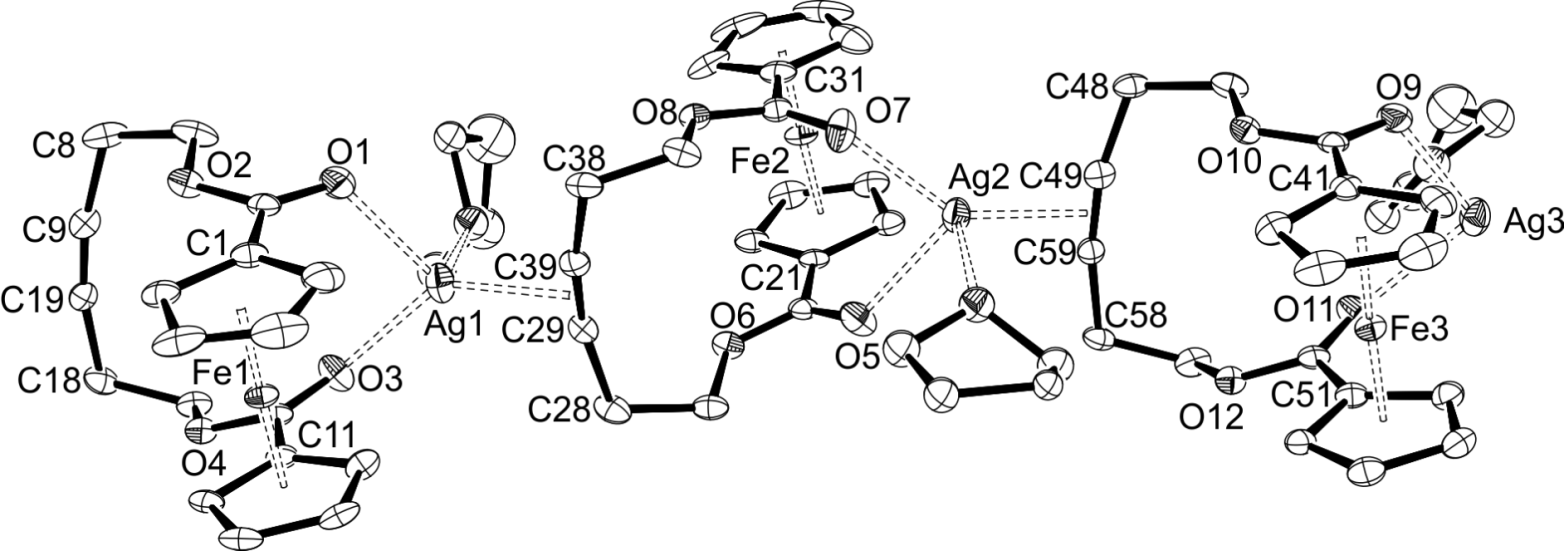
ORTEP diagram of **5**(thf) with thermal displacement drawn at 50% probability; hydrogens atoms, [SbF_6_]^−^ anions are omitted for clarity, only the main positions of the THF molecules are shown. Selected bond lengths [Å] and angles [°] are given in Supporting Information File 1, Tables S1 and S2.

Single crystals of the silver complex **5** suitable for X-ray diffraction analysis could be obtained after layering a THF solution with hexane. The resulting ORTEP drawing is shown in Figure 10. The molecular structure of **5** turned out to be a coordination polymer with a rather loose coordination of the silver towards the ferrocene unit. Compound **5** crystallises in the monoclinic space group *P*2_1_/*c* with three independent molecules per asymmetric unit. The silver atom is on the one hand coordinated by the oxygen atoms of the carbonyl groups from one ferrocenophane, on the other hand through the alkyne moiety of the neighbouring ferrocenophane, and additionally by one THF molecule. The Ag–(C≡C) distances of 2.2321(3) Å, 2.2390(3) Å, and 2.2453(3) Å for the atoms Ag1, Ag2, and Ag3, respectively, are in good agreement with other Ag–alkyne distances in comparable complexes [99–101]. However, the triple bonds themselves show a moderate deviation from linearity with angles between 166.3(5)° (C49–C59) and 168.9(5)° (C9–C19). The ferrocene units show a zigzag pattern if looked from top of one of the cyclopentadienyl rings. The crystallographic data for the ferrocene units is in the same range as for the free ligand **2a**.

## Conclusion

The present paper reports a new application of terminal alkyne metathesis (TAM) using the highly active molybdenum pre-catalyst **MoF6**. For the first time, acetylenic ferrocenophanes were accessed using ring-closing alkyne metathesis. Interestingly, the chain length of the acetylenic functional group plays a great role in the outcome of the metathesis reaction. While catalyst **MoF6** was not able to perform an RCAM on the propargyl-bearing substrate, the ferrocenophane **2a** derived from the butynyl substrate **1a** was obtained in high yields even in concentrated solution. Further increasing the distance between the alkyne moiety and the ester function, thus employing the pentynyl substrate **1b** in an RCAM reaction, a decrease of the selectivity with formation of the monomeric as well as the dimeric ring-closed products was observed. Furthermore, the reactivity of **2a** towards Ag(SbF_6_) was investigated. It was found that this reaction is solvent dependant, and **2a** was readily oxidized in DCM solution to the corresponding ferrocenium hexafluoroantimonate **4**, whereas the silver(I) coordination polymer **5** was isolated from the reaction in THF solution. This behaviour can be ascribed to the fact that Ag(SbF_6_) acts as a stronger oxidant in DCM compared to THF solution [98].

## Supporting Information

CCDC 1870273–1870279 contain the supplementary crystallographic data for this paper. These data can be obtained free of charge via http://www.ccdc.cam.ac.uk/data_request/cif.

File 1 Experimental section, NMR spectra, catalysis procedures and product characterisation, crystallographic data.
