# Supplementary material for: Formation of alkyne-bridged ferrocenophanes using ring-closing alkyne metathesis on 1,1’-diacetylenic ferrocenes
Source: Beilstein J Org Chem. 2019 Oct 24;15:2534–43. doi: 10.3762/bjoc.15.246 (PMC6839559; doi:10.3762/bjoc.15.246)
Supplement: File 1 — Experimental section, NMR spectra, catalysis procedures and product characterisation, crystallographic data. [file Beilstein_J_Org_Chem-15-2534-s001.pdf]

BEILSTEIN JOURNAL OF ORGANIC CHEMISTRY

Supporting Information

for

Formation of alkyne-bridged ferrocenophanes using ring-closing alkyne metathesis on 1,1'-diacetylenic ferrocenes

Celine Bittner, Dirk Bockfeld and Matthias Tamm

Beilstein J. Org. Chem. **2019,** *15,* 2534–2543. doi:10.3762/bjoc.15.246

Experimental section, NMR spectra, catalysis procedures and product characterisation, crystallographic data

License and Terms: This is a supporting information file under the terms of the Creative Commons Attribution License (http://creativecommons.org/licenses/by/4.0). Please note that the reuse, redistribution and reproduction in particular requires that the authors and source are credited.
The license is subject to the *Beilstein Journal of Organic Chemistry* terms and conditions: (https://www.beilstein-journals.org/bjoc)

Contents

1. General information S1
2. Experimental section S2
   2.1. General procedure for the synthesis of ferrocenyl esters **1a** and **1b** S2
   2.2. Catalytic ring-closing alkyne metathesis S3
   2.3. Synthesis of [Fe(III){Cp(COO(CH_2)$_2$)}$_2$C≡C][SbF_6] (**4**) S5
   2.4. General procedure for the complexation of ferrocenophane **2a** S5
3. NMR Spectra S7
4. Crystallographic data S14
5. References S20

1. General Information

Experimental. All operations with air and moisture-sensitive compounds were conducted in a glovebox (MBraun 200B) under a dry and oxygen-free argon atmosphere or on a Schlenk line using standard Schlenk techniques. The herefore exploited argon gas 4.6 (Linde AG und Westfalen AG) was passed over BTS-catalyst (BASF) at 130 °C for oxygen removal and over Sicapent (phosphorous pentoxide with colour indicator, VWR) for the removal of water. Solvents were purified and dried by an mBraun Solvent Purification System (SPS) and stored over molecular sieve (3−4 Å). Complex **MoF6** and ferrocenyl dichloride **3** were synthesized according to literature methods.[1,2] All other compounds were obtained from commercial sources and used without further purification. Flash chromatography was performed on silica gel (230–400 mesh) from Merck in combination with HPLC grade solvents. The powdered molecular sieve with a pore size of 5 Å (Sigma-Aldrich, powder <50 µm) for metathesis reactions (MS 5 Å) was dried for 24 h at 180 °C under vacuum prior to use.

Analytical methods. ^{1}H and ^{13}C NMR spectra were recorded on Bruker AV II-300, DRX-400, and AV II-600 instruments at room temperature. Chemical shifts (δ) are expressed in ppm (parts per million). and are referenced to residual solvent signals ($CDCl_3$: δ(H) 7.26 ppm, δ(C) 77.16 ppm; CD_2Cl_2: δ(H) 5.32 ppm, δ(C) 53.84 ppm; THF-d_8: δ(H) 1.72 ppm and 3.58 ppm, δ(C) 25.31 ppm and 67.21 ppm). Coupling constants (J) are reported in hertz (Hz). Multiplicities are expressed with s (singlet), d (doublet), t (triplet), q (quartet), or m (multiplet). The number of hydrogen atoms (n) for a signal is indicated by nH. When necessary, signal assignment was confirmed by two-dimensional H,C-HSQC and H,C-HMBC NMR experiments. Elemental analyses (TU Braunschweig) were accomplished by combustion and gas chromatographic analysis using a VarioMICRO Tube and WLD detection. ESI mass spectrometry (TU Braunschweig) in MeOH was performed on an LTQ-Orbitrap Velos linear iontrap with orbitrap mass

analyser (Thermo Fisher Scientific, Bremen, Germany) in direct infusion mode using a custom made microspray-device mounted on a Proxeon nanospray ion source. The microspray-device allows for the sample infusion through a stainless steel capillary (90 µm i. d.). Accurate mass measurements in the orbitrap were performed using the lock mass option of the instrument control software using the cation of tetradecyltrimethylammonium bromide (0.1 mg/mL; 256.29988 amu) as internal mass reference.

2. Experimental Section

2.1. General Procedure for the synthesis of ferrocenyl esters 1a and 1b

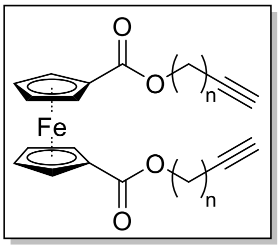

To a solution of the alcohol (2.1 equiv, 13.5 mmol), NEt_3 (2.1 equiv, 13.5 mmol), and DMAP (0.08 equiv, 0.5 mmol) in DCM (20 mL) at 0 °C a solution of ferrocenyl dichloride **3** (2 g, 6.4 mmol) in DCM (20 mL) is slowly added via a dropping funnel under inert gas atmosphere. The resulting orange suspension is stirred overnight at room temperature. The reaction mixture is then washed with water and sat. NaCl (aq), dried over Na_2SO_4 and evaporated under reduced pressure. The dark brown and oily crude product is purified using column chromatography (hexane/EtOAc 1:5). The products **1a** and **1b** are obtained as crystalline orange solids. The NMR spectroscopic data for **1a** fit the published literature.[3]

1a *n* = 2 yield 2 g, 82%.

^{1}H NMR (200.1 MHz, $CDCl_3$, 298 K): δ [ppm] = 4.85 (m, 2H, CH_β), 4.43 (m, 2H, CH_α), 4.32 (t, $^3J_{HH}$ = 6.7 Hz, 2H, OCH_2), 2.64 (td, $^3J_{HH}$ = 6.7 Hz, $^4J_{HH}$ = 2.7 Hz, 2H, CH_2C≡CH), 2.05 (t, $^4J_{HH}$ = 2.7 Hz, 1H, C≡C*H*).

^{13}C{^{1}H} NMR (50.3 MHz, $CDCl_3$, 298 K): δ [ppm] = 170.3 (s, *C*=O), 80.5 (s, *C*≡CH), 73.2 (s, 2 *C*H_β), 72.6 (s, *ipso–C*), 71.8 (s, 2 *C*H_α), 70.0 (s, C≡*C*H), 62.3 (s, O*C*H_2), 19.3 (s, *C*H_2C≡CH).

Elemental analysis (%) calcd for $C_{20}H_{18}FeO_4$: C 63.52, H 4.80; found C 63.96, H 4.85.

1b *n* = 3 yield 2.46 g, 94%.

^{1}H NMR (200.1 MHz, $CDCl_3$, 298 K): δ [ppm] = 4.83 (m, 2H, CH_β), 4.42 (m, 2H, CH_α), 4.33 (t, $^3J_{HH}$ = 6.3 Hz, 2H, OCH_2), 2.39 (td, $^3J_{HH}$ = 7.0 Hz, $^4J_{HH}$ = 2.7 Hz, 2H, CH_2C≡CH), 2.01 (t, $^4J_{HH}$ = 2.7 Hz, 1H, C≡C*H*), 2.01-1.92 (m, 2H, CH_2CH_2C≡CH).

^{13}C{^{1}H} NMR (50.3 MHz, $CDCl_3$, 298 K): δ [ppm] = 170.5 (s, *C*=O), 83.2 (s, *C*≡CH), 73.0 (s, 2 *C*H_β), 72.9 (s, *ipso–C*), 71.6 (s, 2 *C*H_α), 69.3 (s, C≡*C*H), 63.1 (s, O*C*H_2), 27.8 (s, OCH_2*C*H_2), 15.4 (s, *C*H_2C≡CH).

elemental analysis (%) calcd for $C_{22}H_{22}FeO_4$: C 65.04, H 5.46; found C 64.54, H 5.52.

2.2. Catalytic ring-closing alkyne metathesis

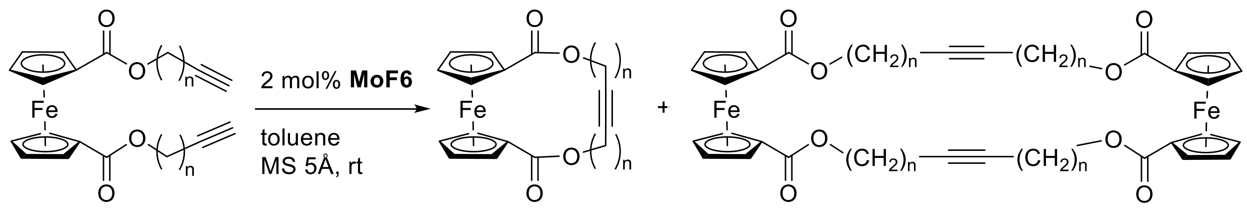

1a n=2
1b n=3

2a n=2
2b n=3

S1 n=2
S2 n=3

Scheme S1. Catalytic RCAM of substrates **1a** and **1b** towards the monomeric ferrocenophanes **2a** and **2b**; in some cases the formation of the dimeric ferrocenophanes **S1** and **S2** were observed by means of NMR spectroscopy and mass spectrometry.

Preparation of ferrocenophane 2a

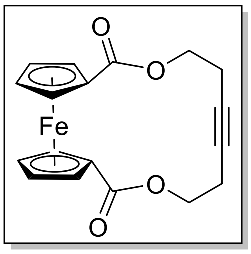

To a solution of **1a** (0.25 mmol) and MS 5Å in toluene (56 mL, 4.5 mM) under inert Ar atmosphere the catalyst **MoF6** (2 mol %, 0.005 mmol) is added as a solid. After stirring at room temperature for 2 hours the reaction mixture is filtered over silica and the patch is washed with Et_2O and DCM. The orange filtrate is evaporated under reduced pressure and the crude product was purified by flash column chromatography on silica gel with 1:5 hexane/EtOAc yielding **2a** as an orange crystalline powder. In a second attempt the reaction was repeated using 12 mL of toluene (21 mM). Crystals suitable for X-ray diffraction analysis could be obtained from a slowly cooled (−28 °C) saturated solution of **2a** in toluene.

yield: 93% (4.5 mM), 92% (21 mM).

^{1}H NMR (300.3 MHz, $CDCl_3$, 298 K): δ [ppm] = 4.75 (m, 2H, CH_β), 4.51 (m, 2H, CH_α), 4.24 (m, 2H, OCH_2), 2.59 (m, 2H, CH_2C≡C).

^{13}C{^{1}H} NMR (75.5 MHz, $CDCl_3$, 298 K): δ [ppm] = 171.0 (s, *C*=O), 78.5 (s, *C*≡*C*), 73.6 (s, *iC*), 72.2 (s, *C*H_β), 71.8 (s, *C*H_α), 63.7 (s, O*C*H_2), 19.2 (s, *C*H_2C≡C).

elemental analysis (%) calcd for $C_{18}H_{16}FeO_4$: C 61.39, H 4.58; found C 61.87, H 4.70.

When the reaction takes place in further concentrated solution (125 mM) four species can be identified with the help of mass spectrometry. Starting material **1a** exact mass theoretical *m/z* = 387.01 ($C_{20}H_{18}FeO_4$), found: 378.1; monomeric ring-closed product **2a** exact mass theoretical *m/z* = 352.04 ($C_{18}H_{16}FeO_4$), found: 352.0; ring-closed dimer **S1** exact mass theoretical *m/z* = 704.08 ($C_{36}H_{32}Fe_2O_8$), found: 704.8; open dimer exact mass theoretical *m/z* = 730.10 ($C_{38}H_{34}Fe_2O_8$), found: 730.0.

Preparation of ferrocenophane 2b

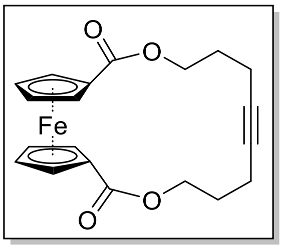

To a solution of **1b** (0.25 mmol) and MS 5 Å in toluene (56 mL, 4.5 mM) under inert Ar atmosphere the catalyst **MoF6** (2 mol %, 0.005 mmol) is added as a solid. After stirring at room temperature for 4 hours the reaction mixture is filtered over silica and the patch is washed with Et_2O and DCM. The orange filtrate is evaporated under reduced pressure and the crude product (42 mg, 89%) was purified by flash column chromatography on silica gel with 5:1 hexane/EtOAc yielding 25 mg (53%) of **2b** as an orange crystalline powder. The dimeric compound **S2** could only be eluted as a mixture with **2b** in a ratio of roughly 1:0.75 (16 mg). However, single crystals of **2b** suitable for X-ray analysis could be obtained from that mixture from a DCM solution layered with *n*-hexane.

^{1}H NMR (300.3 MHz, $CDCl_3$, 298 K): δ [ppm] = 4.81 (m, CH_β), 4.44 (m, CH_α), 4.43 (t, $^3J_{HH}$ = 6.0 Hz, OCH_2) 2.41 (m, CH_2C≡C), 1.92 (m, CH_2CH_2C≡C).

^{13}C{^{1}H} NMR (75.5 MHz, $CDCl_3$, 298 K): δ [ppm] = 170.5 (s, *C*=O), 80.8 (s, *C*≡*C*), 74.0 (s, *iC*), 72.0 (s, *C*H_β), 71.8 (s, *C*H_α), 62.9 (s, O*C*H_2), 26.8 (s, *C*H_2CH_2C≡C), 15.9 (s, *C*H_2C≡C).

MS: exact mass theoretical *m/z* = 403.06032 ($C_{20}H_{20}FeO_4Na$); found: 403.06041.

NMR characterization of the side product **S2**

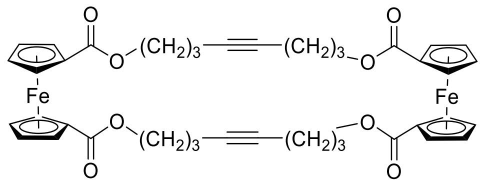

S2

^{1}H NMR (300.3 MHz, $CDCl_3$, 298 K): δ [ppm] = 4.76 (m, CH_β), 4.44-4.40 (m, CH_α, overlay with CH_α & OCH_2 of **2b**), 4.32 (t, $^2J_{HH}$ = 6.2 Hz, OCH_2), 2.43-2.35 (m, CH_2C≡C, overlay with the corresponding signal of **2b**), 1.95-1.88 (m, CH_2CH_2C≡C, overlay with the corresponding signal of **2b**).

^{13}C{^{1}H} NMR (75.5 MHz, $CDCl_3$, 298 K): δ [ppm] = 170.4 (s, *C*=O), 79.8 (s, *C*≡*C*), 73.2 (s, *iC*), 72.6 (s, *C*H_β), 71.8 (s, *C*H_α, overlay with the corresponding signal of **2b**), 63.3 (s, O*C*H_2), 28.2 (s, *C*H_2CH_2C≡C), 15.7 (s, *C*H_2C≡C).

MS: exact mass theoretical *m/z* = 783.13139 ($C_{40}H_{40}Fe_2O_8Na$); found: 783.13189.

2.3. Synthesis of [Fe(III){Cp(COO(CH2)2)}2C≡C][SbF6] (4)

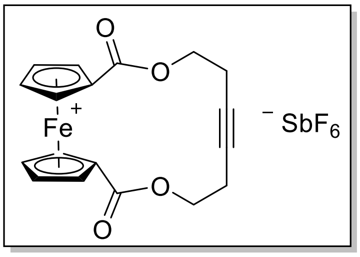

To a solution of the ferrocenophane **2a** (30 mg, 0.085 mmol) in DCM (3 mL) is added a solution of Ag(SbF6) (29.3 mg, 0.085 mmol) in DCM (2 mL) while stirring. An immediate colour change from orange to dark green can be observed. After 16 hours at room temperature the reaction mixture is filtered over silica and the solvent is evaporated under reduced pressure. Crystallisation with hexane from a saturated solution of the crude product in DCM affords the ferrocenium 4 as dark blue crystalline needles.

yield: 48 mg, 96%.

^{1}H NMR (300.2 MHz, CD_2Cl_2, 298 K): δ [ppm] = 1.37-1.29 (m, C*H*), 0.90 (t, $^3J_{HH}$ = 6.8 Hz, CH_2), —3.80 (br s, CH_{Cp}), —5.43 (m, CH_{Cp}).

elemental analysis (%) calcd for $C_{18}H_{16}F_6FeO_4Sb$: C 36.77, H 2.74; found C 36.51, H 2.917.

2.4. General procedure for the complexation of ferrocenophane 2a

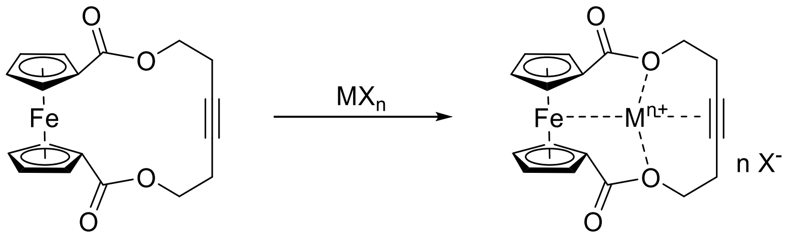

2a **2a/M**

Scheme S2. Attempted complexation of transition metals with ferrocenophane **2a**.

The transition metal salt (0.057 mmol) is solved or suspended in THF (1 mL). A solution of **2a** (20 mg, 0.057 mmol) in THF (1 mL) is added. After 16 hours at room temperature the orange suspension is filtered over a short patch of celite and the solvent is evaporated in vacuum.

2a/Pd: $[(MeCN)_4Pd][BF_4]_2$ (25.2 mg) was used as a Pd source. The resulting **2a/Pd** can be obtained with precipitation after addition of hexane from a saturated solution of **2a/Pd** in DCM as a light brown powder.

yield: 27 mg.

^{1}H NMR (200.1 MHz, $CDCl_3$, 298 K): δ [ppm] = 4.99-4.29 (m, 4H, CH_{Cp}), 3.79 (br s, 2H, OCH_2), 3.42 (m, thf), 1.90 (br s, 2H, CH_2C≡C), 1.63 (m, thf).

2a/Ag: Ag(SbF_6) (19.5 mg) was used as a Ag source. The coordination polymer **5**(thf) can be obtained with hexane from a saturated solution of **5**(thf) in THF as orange crystals.

yield: 34 mg.

^{1}H NMR (200.1 MHz, thf-d_8, 298 K): δ [ppm] = 4.90 (m, 2H, CH_{Cp}), 4.72 (m, 2H, CH_{Cp}), 4.37 (m, 2H, OCH_2), 3.80 (br m, thf), 2.77 (m, 2H, CH_2C≡C), 1.95 (br m, thf).

2a/Au: (Me_2S)AuCl (16.7 mg) was used as a Au source. The reaction is performed under the exclusion of light. The resulting **2a/Au** can be obtained as a light orange powder after evaporation of the solvent. Crystallisation from a saturated solution in THF layered with Et_2O afforded orange crystals suitable for X-ray diffraction analysis. However, these crystals turned out to be a polymorph of the of the free ligand **2b**.

yield: 19 mg.

^{1}H NMR (200.1 MHz, thf-d_8, 298 K): δ [ppm] = 4.67 (br s, 2H, CH_{Cp}), 4.49 (br s, 2H, CH_{Cp}), 4.13 (m, 2H, OCH_2), 2.53 (m, 2H, CH_2C≡C).

3. NMR Spectra

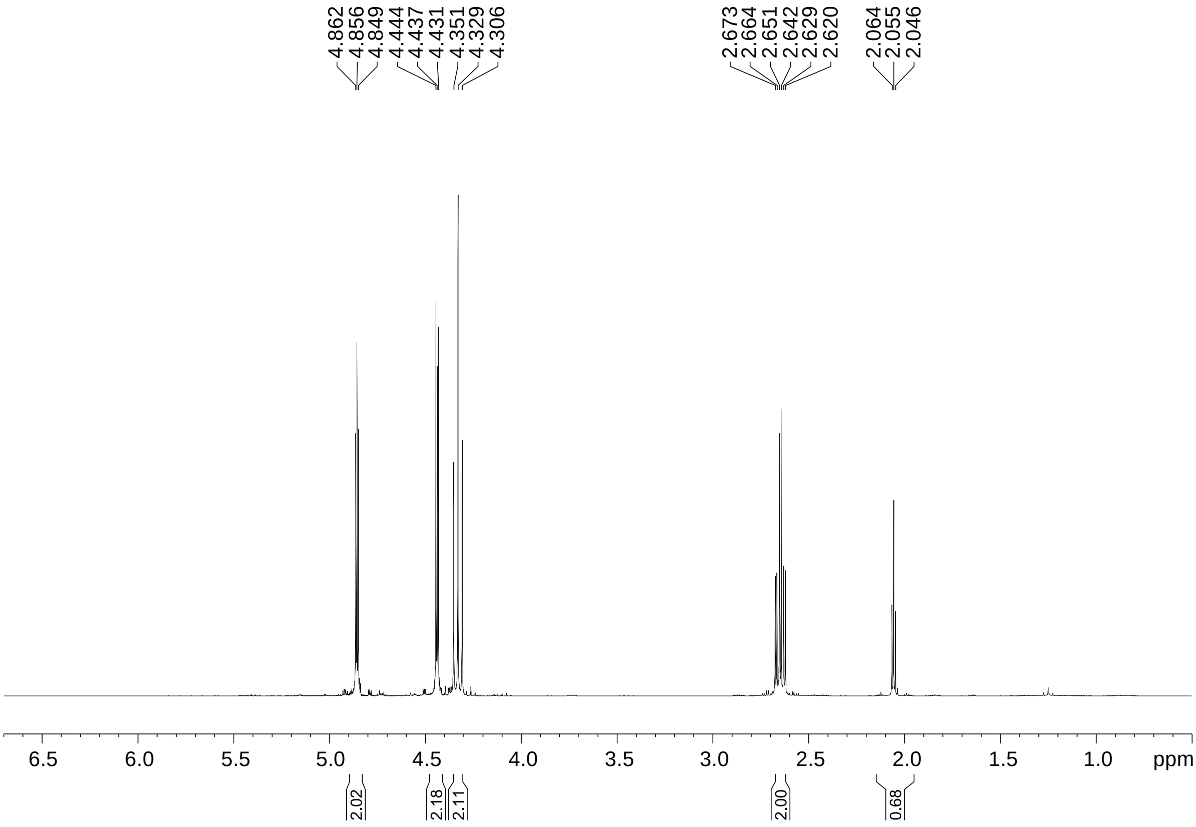

Figure S1. ^{1}H NMR spectrum (300.3 MHz, $CDCl_3$, 298 K) of butinyl ferrocene diester **1a**.

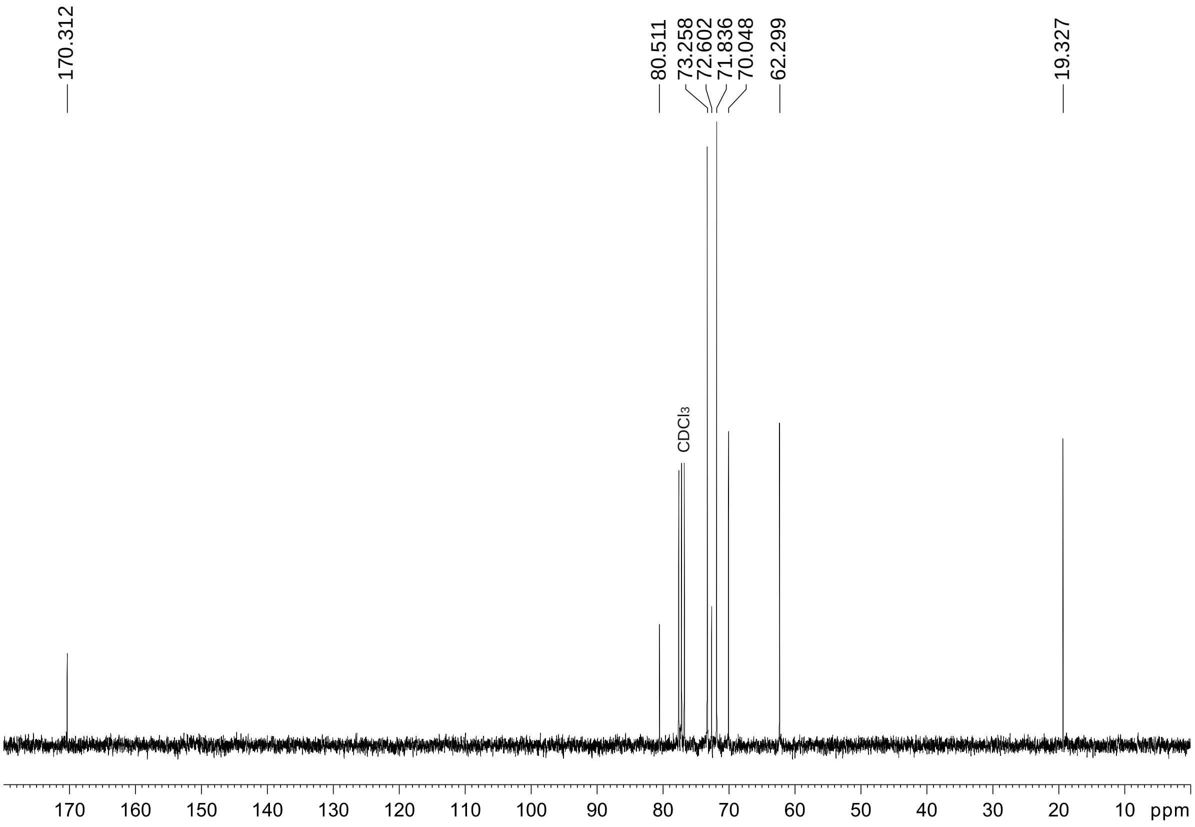

Figure S2. : ^{13}C NMR spectrum (75.5 MHz, $CDCl_3$, 298 K) of butinyl ferrocene diester **1a**.

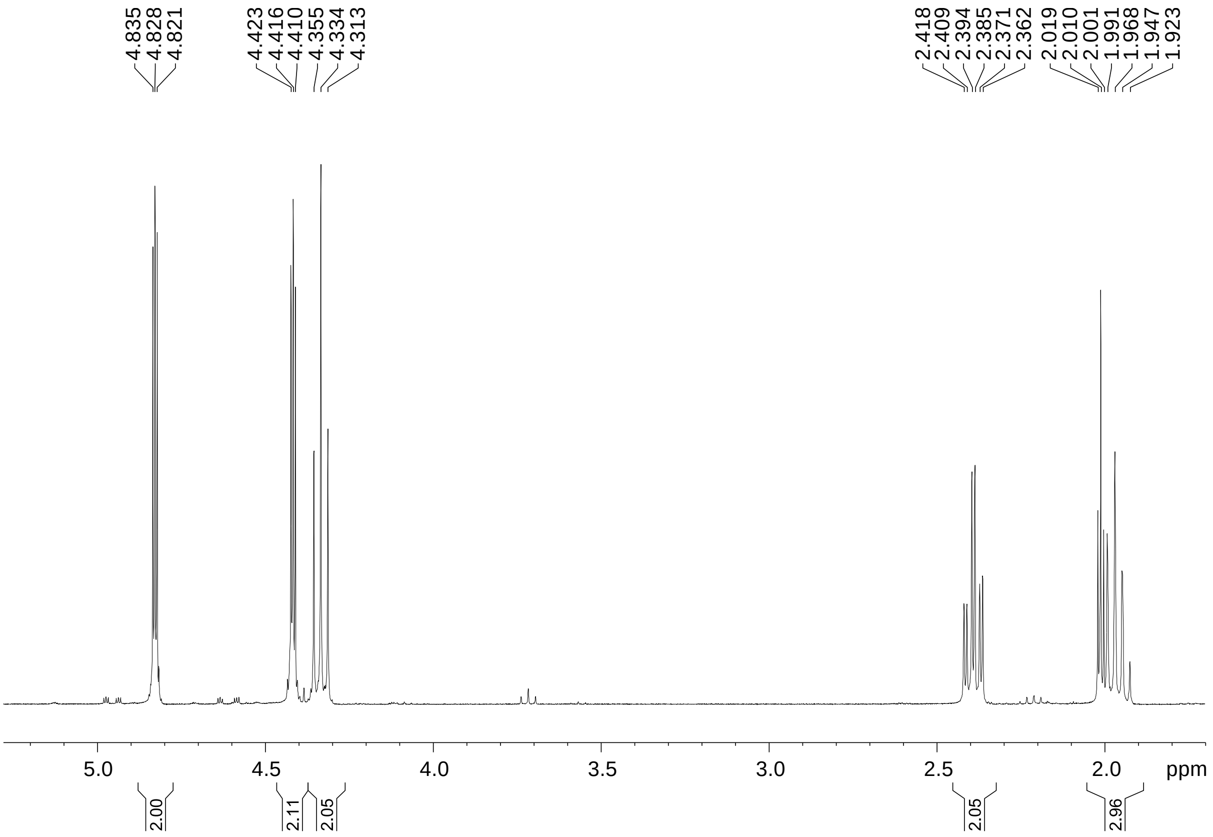

Figure S3. ^{1}H NMR spectrum (300.1 MHz, $CDCl_3$, 298 K) of pentinyl ferrocene diester **1b**.

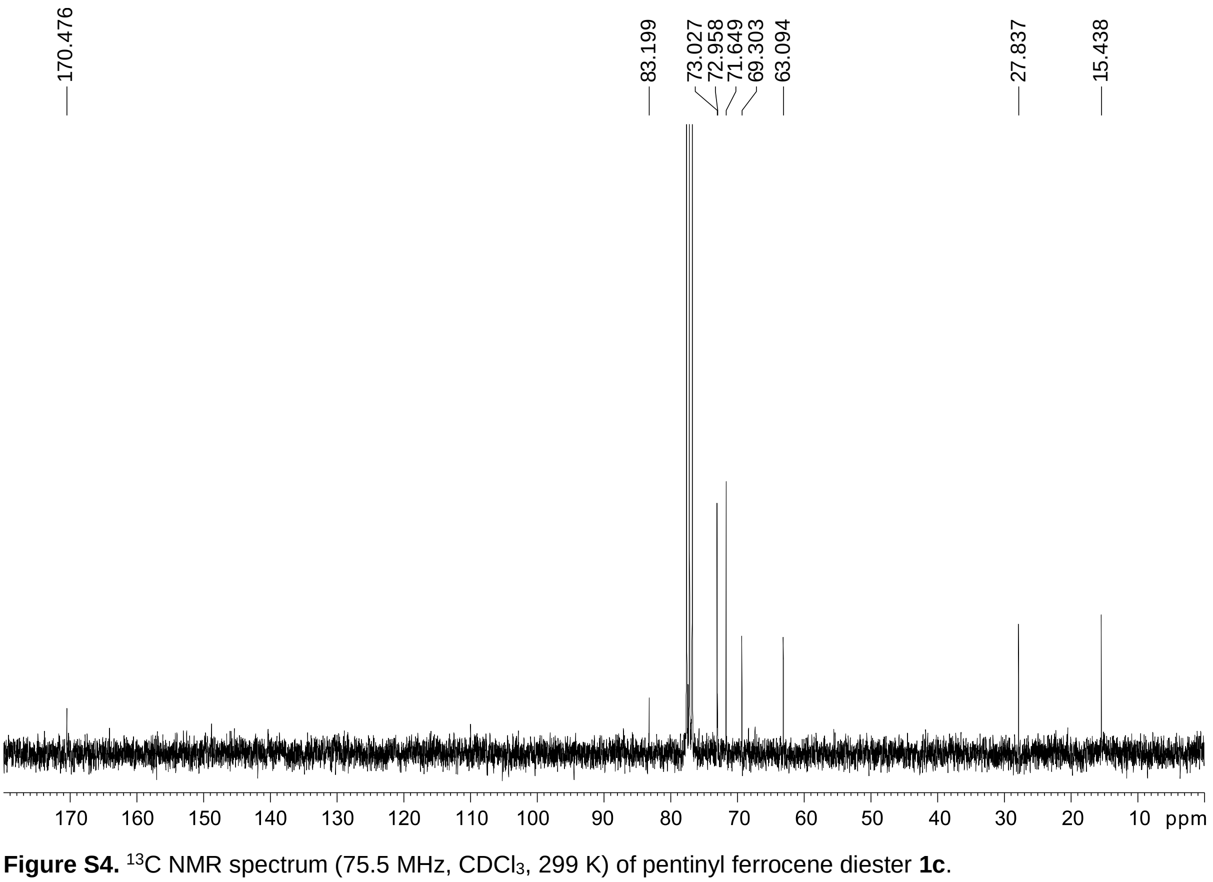

Figure S4. ^{13}C NMR spectrum (75.5 MHz, $CDCl_3$, 299 K) of pentinyl ferrocene diester **1c**.

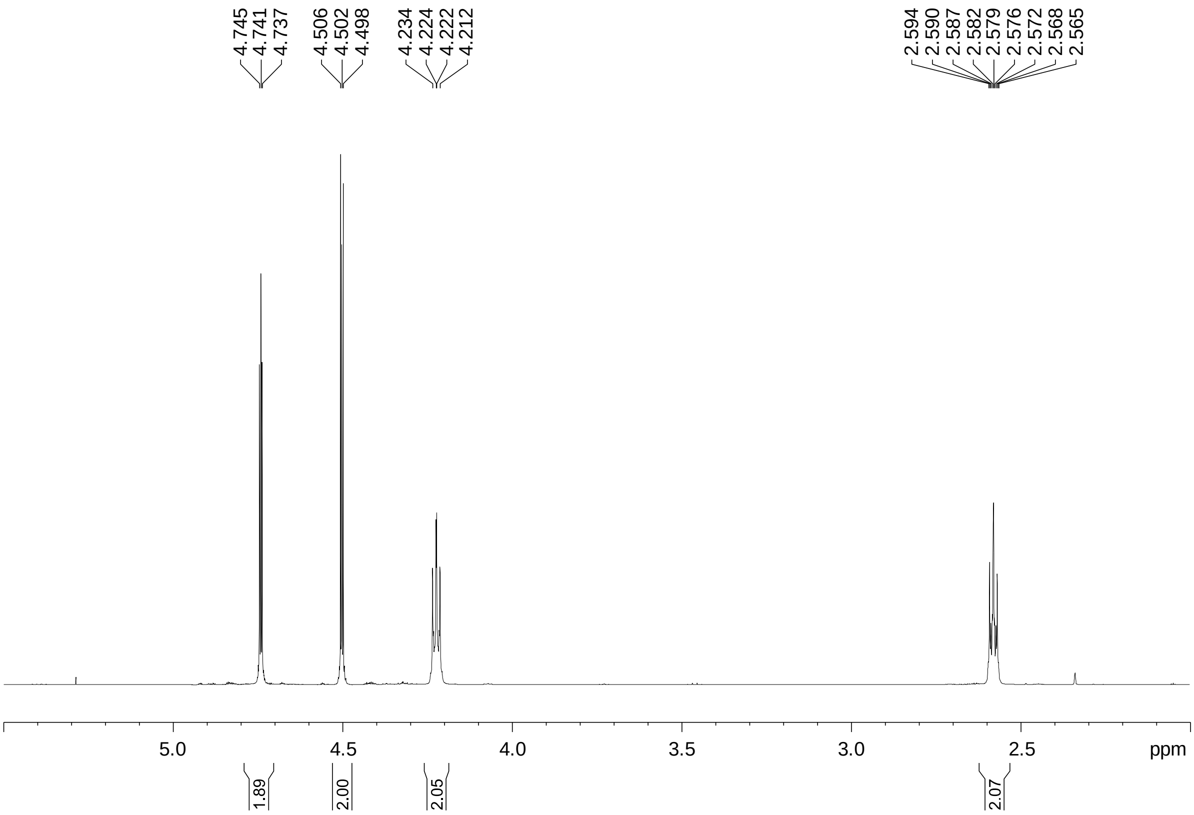

Figure S5. ^{1}H NMR spectrum (500.3 MHz, $CDCl_3$, 298 K) of [10]ferrocenophane **2a**.

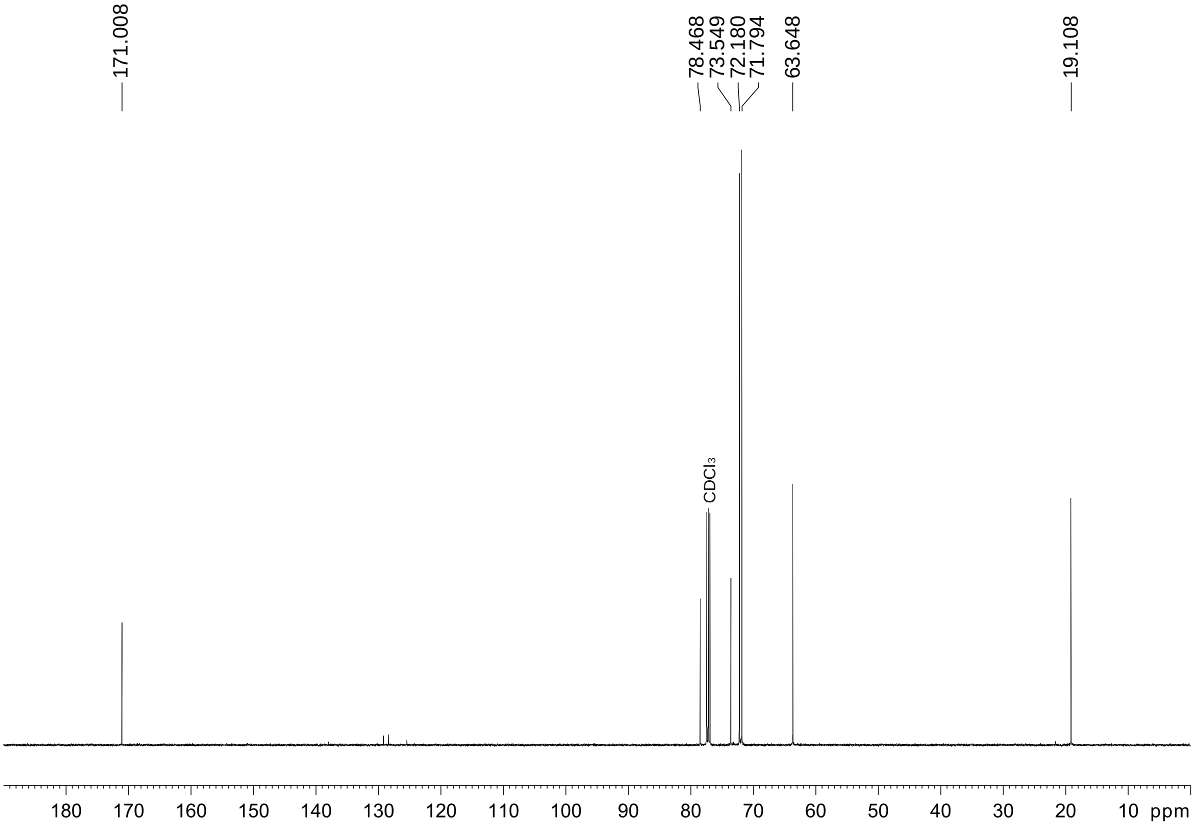

Figure S6. ^{13}C NMR spectrum (125.8 MHz, $CDCl_3$, 298 K) of [10]ferrocenophane **2a**.

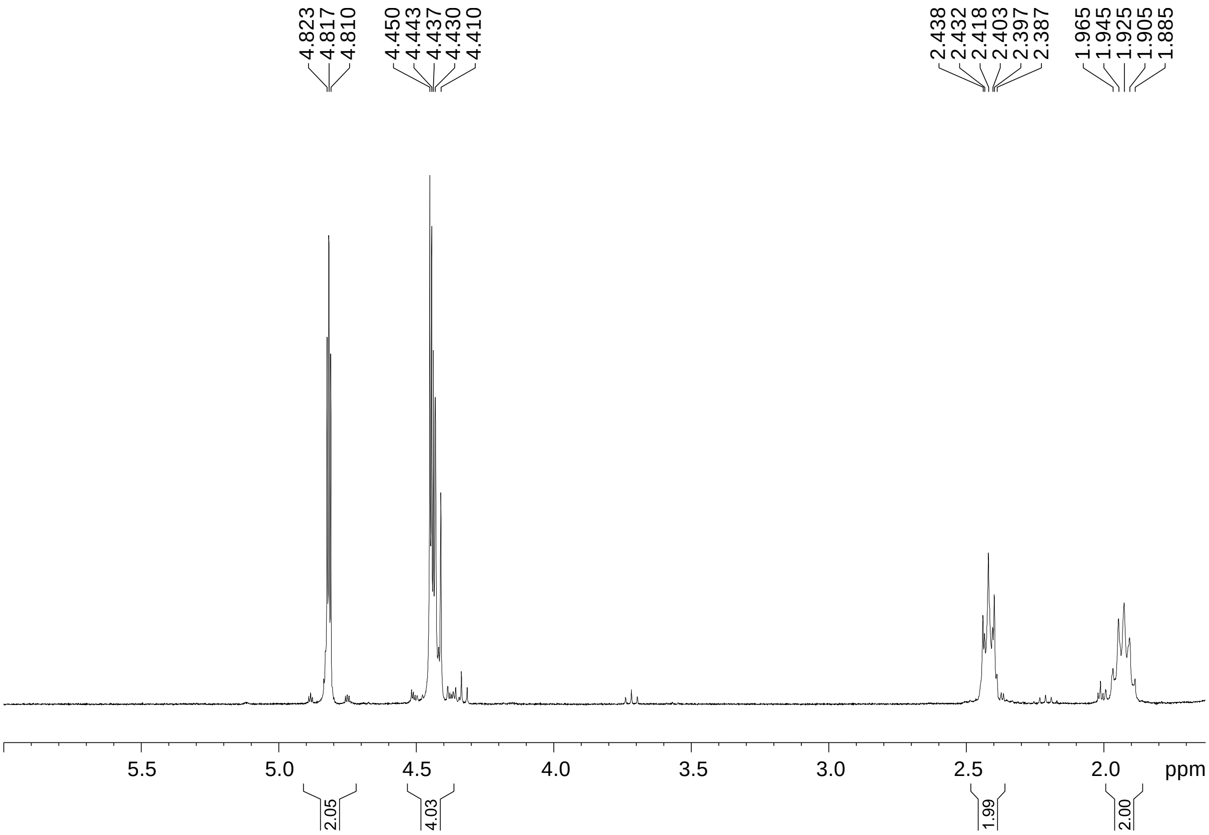

Figure S7. ^{1}H NMR spectrum (300.3 MHz, $CDCl_3$, 298 K) of [12]ferrocenophane **2b**.

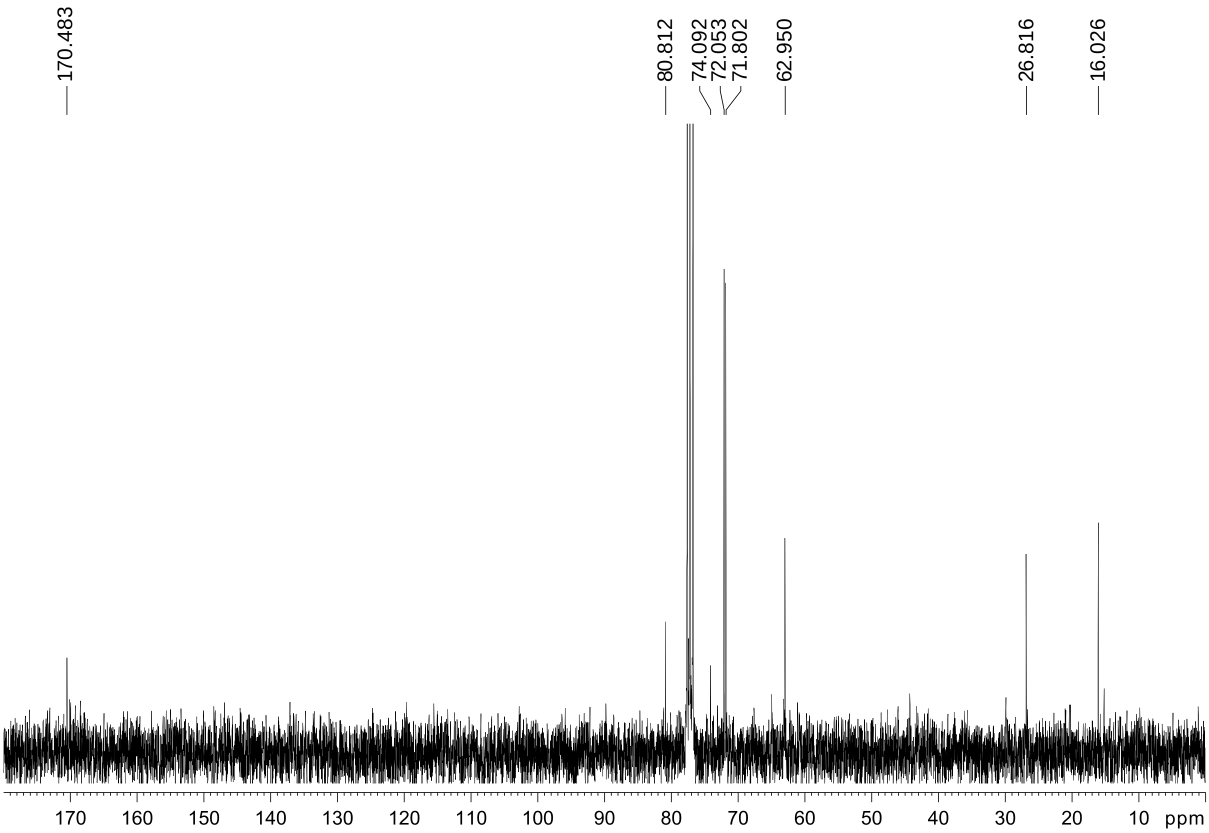

Figure S8. ^{13}C NMR spectrum (125.8 MHz, $CDCl_3$, 298 K) of [12]ferrocenophane **2b**.

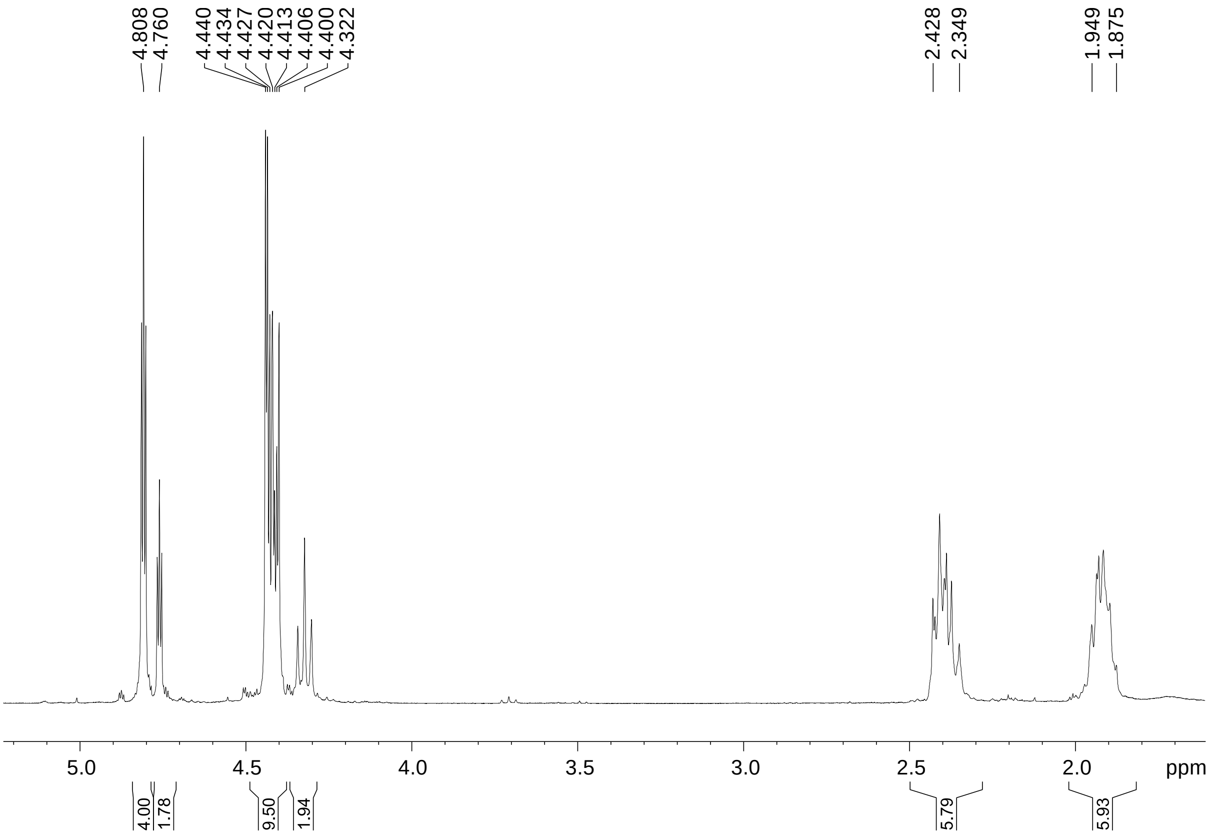

Figure S9. ^{1}H NMR spectrum (300.3 MHz, $CDCl_3$, 298 K) of the crude product obtained after 4 hours from the RCAM of **1b** (0.25 mmol) with **MoF6** (2 mol %) in toluene (24 mL) in the presence of MS 5 Å (500 mg), signals of desired product **2b** overlay with side product **S2**.

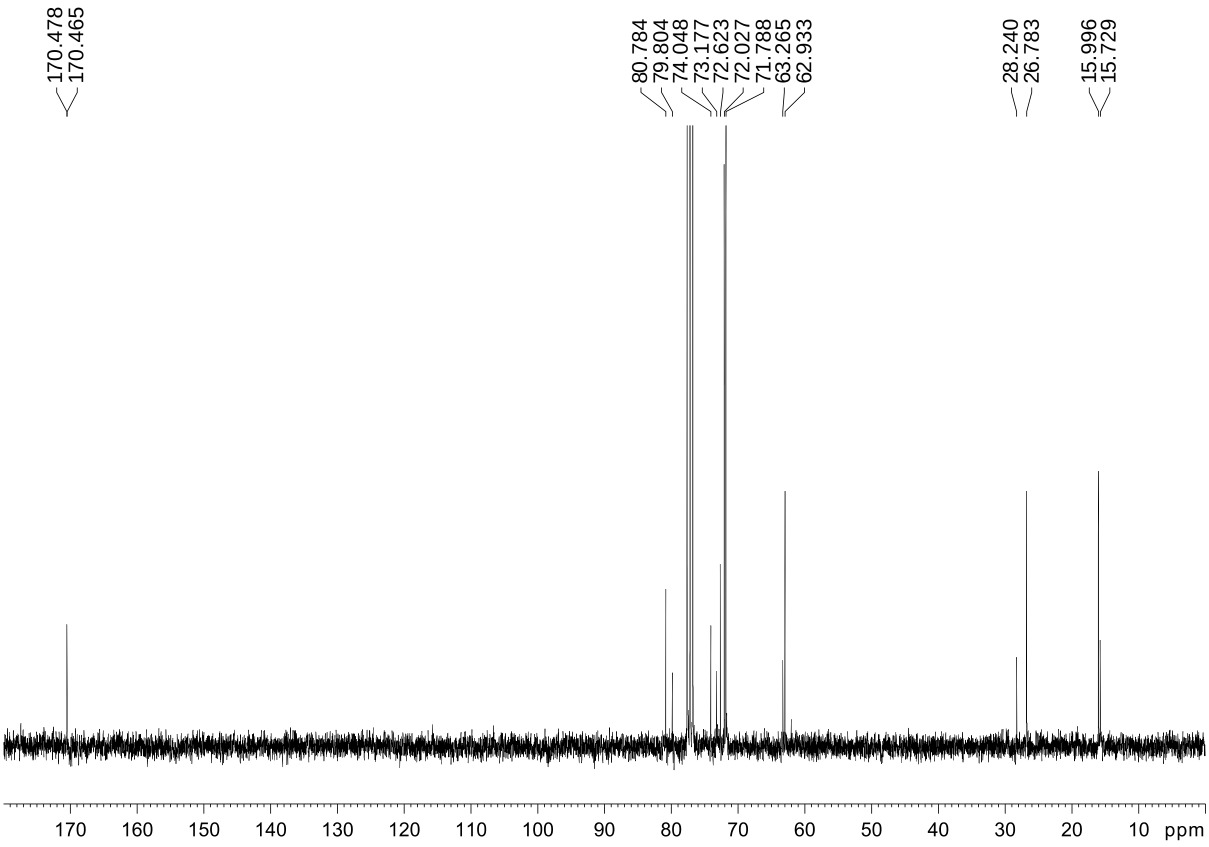

Figure 10. ^{13}C NMR spectrum (75.5 MHz, $CDCl_3$, 298 K) of the crude product obtained after 4 hours from the RCAM of **1b** (0.25 mmol) with **MoF6** (2 mol %) in toluene (24 mL) in the presence of MS 5 Å (500 mg); signals of desired product **2b** overlay with side product **S2**.

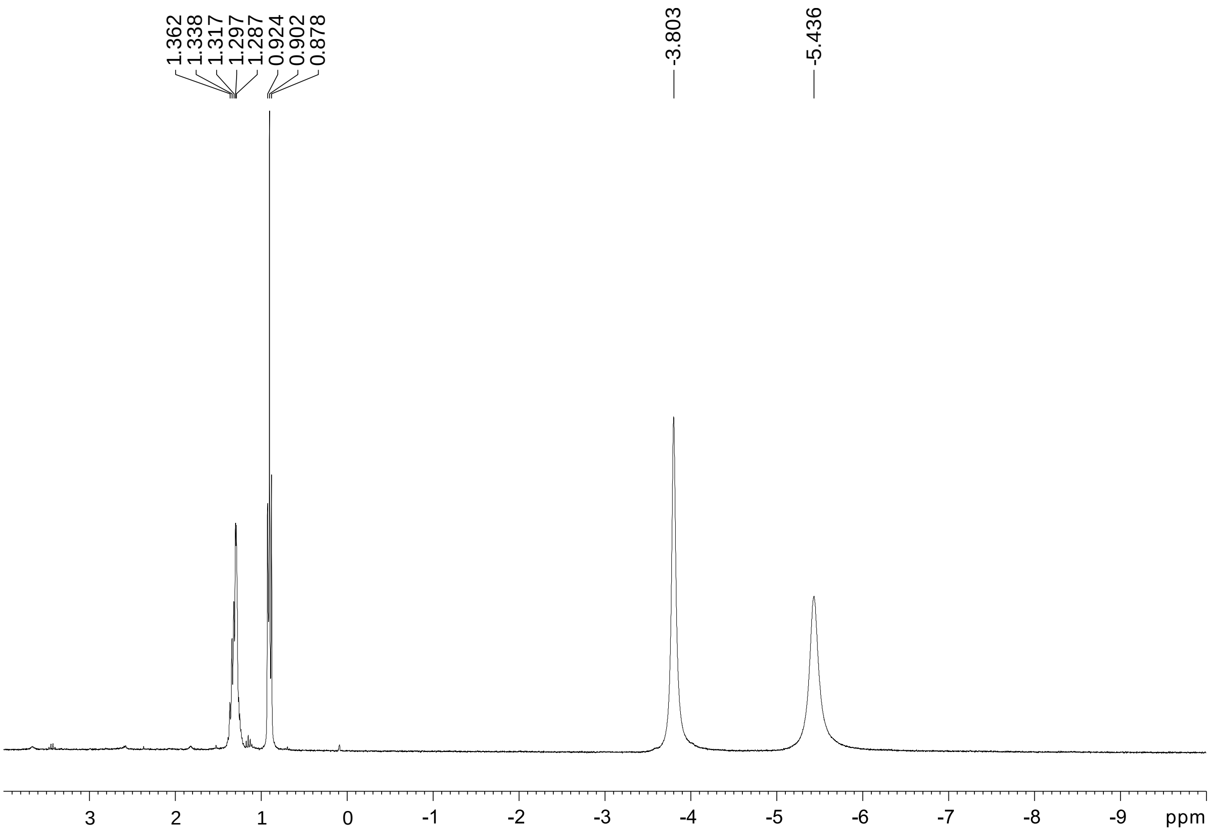

Figure S11. ^{1}H NMR spectrum (300.3 MHz, $CDCl_3$, 298 K) of the ferrocenium **4**.

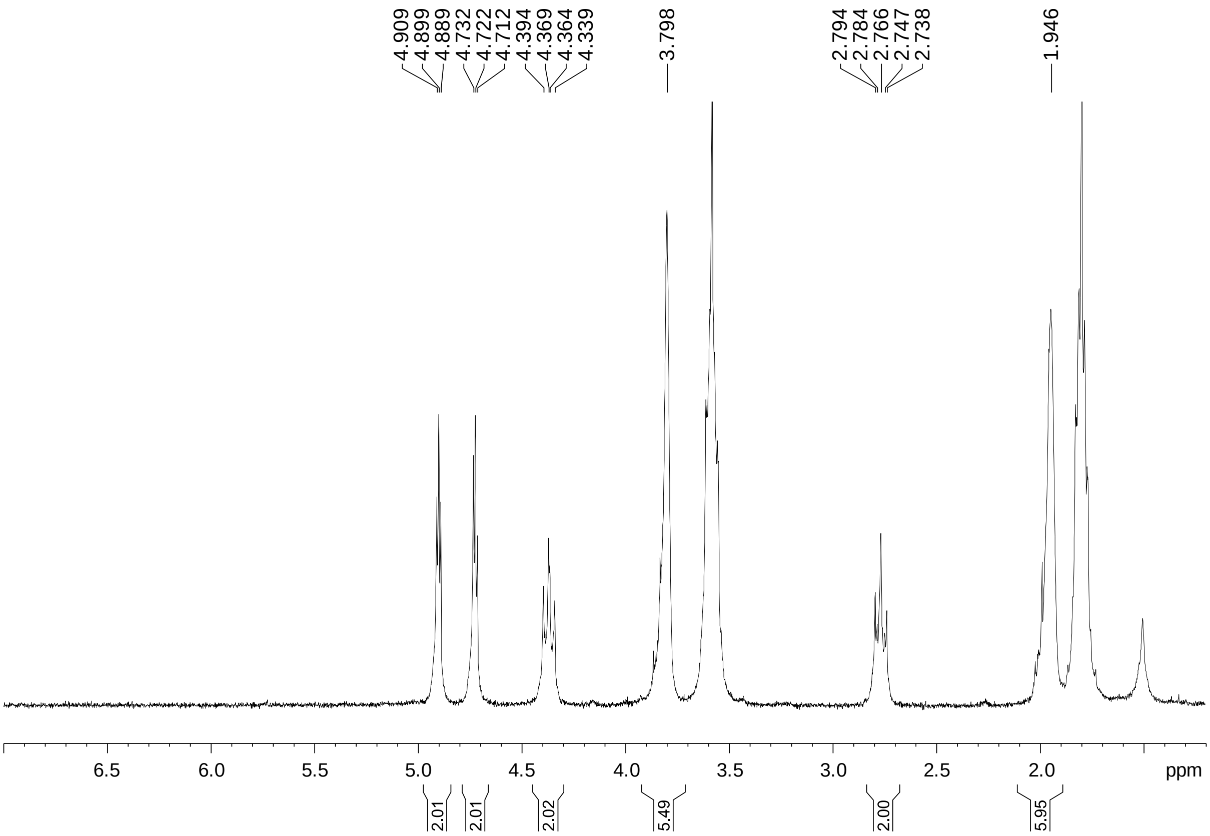

Figure S12. ^{1}H NMR spectrum (200.1 MHz, THF-d_8, 300 K) of coordination polymer **5**.

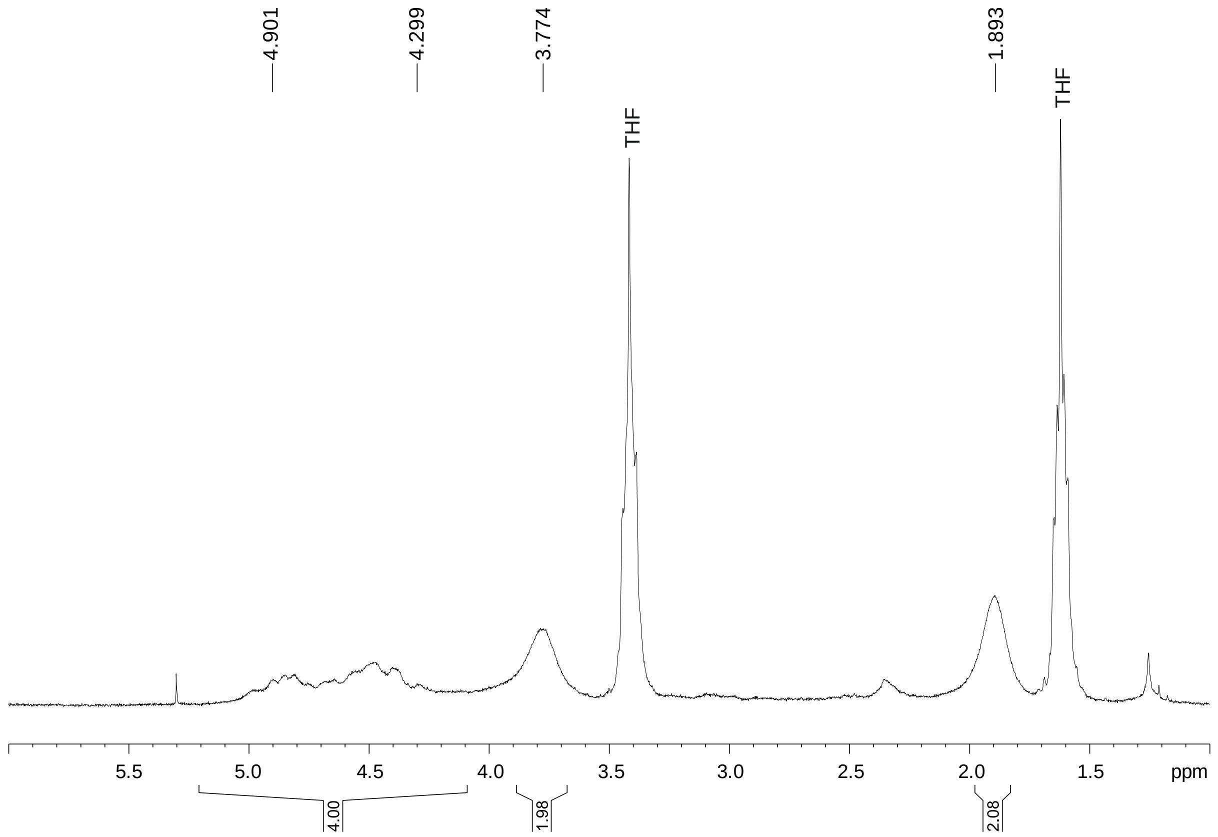

Figure S13. ^{1}H NMR spectrum (200.1 MHz, $CDCl_3$, 300 K) of **2a/Pd**.

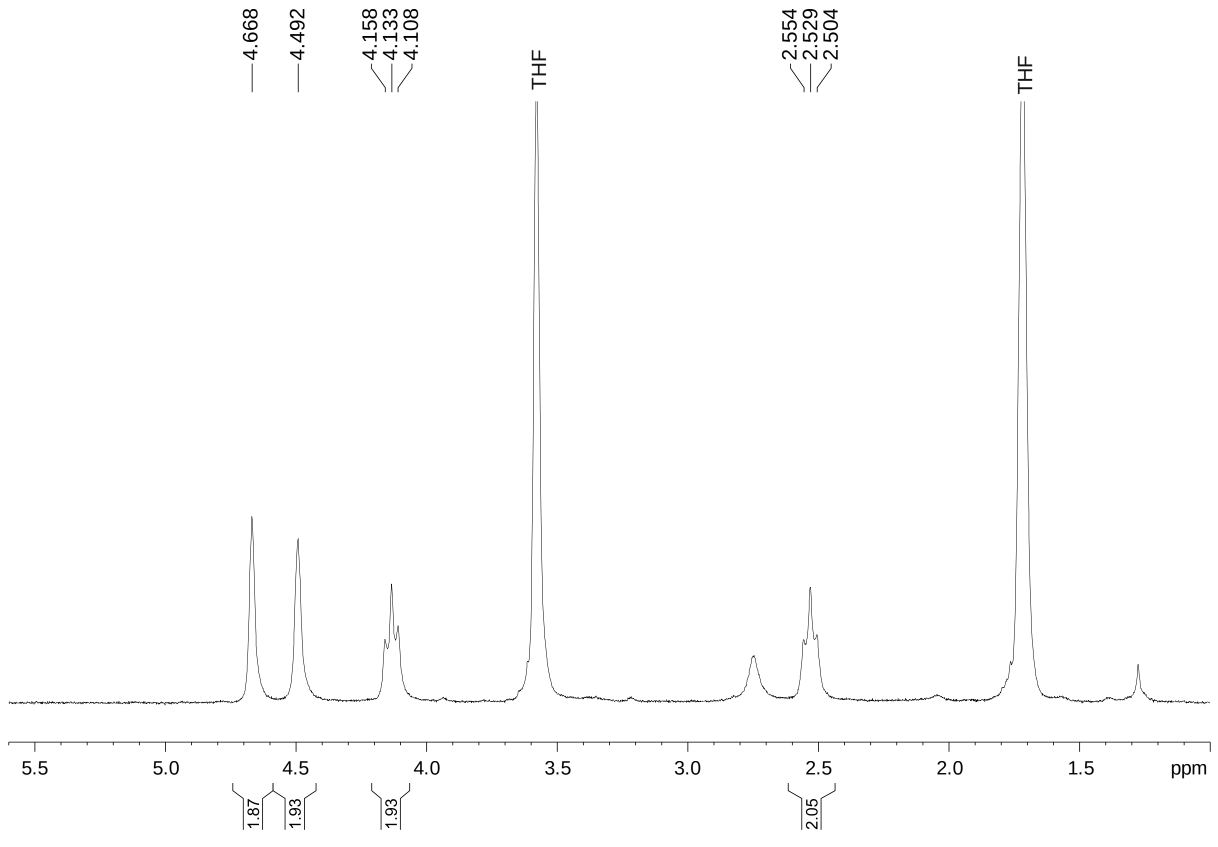

Figure S14. ^{1}H NMR spectrum (200.1 MHz, thf-d_8, 300 K) of **2a/Au**.

4. Crystallographic Data

Numerical data for the coordination polymer **5** are collected in Tables S1 and S2. Crystallographic data for all structures are given in Tables S3–S5. Single crystals were mounted on glass fibers, MiTeGen mounts or on top of a human hair in perfluorinated inert oil. Measurements were performed on Oxford Diffraction Xcalibur diffractometers using monochromated MoKα (**2a°**) or mirror-focused CuKα (**1a**, **1b**, **2a^{a}**, **2b**, **5**) radiation. Data collection of the ferrocenium **4** was performed during a product presentation of Bruker ASX in Karlsruhe by Dr. Holger Ott on a Bruker D8 QUEST diffractometer with mirror-focused MoKα radiation. Data reduction was performed with CrysAlisPro[4] or in case of compound **4** with Bruker Saint.[5] Absorption correction was based on multi-scans. The structures were solved by direct methods with SHELXS-97[6] or intrinsic phasing with SHELXT-2014/5[7] and refined on *F2* using the program SHELXL-2014[6] in OLEX2.[8] H atoms were placed in idealized positions and refined using a riding model.

Special features and exceptions:

1a: The crystal structure was refined as a 2-component inversion twin with a twin ratio of 38% for the minor component. The iron atom was found disordered over two positions with an occupancy of 74% for the major position. The two positions of the iron atoms would correspond to a b-glide plane. Since the occupancy of these two atoms is far from 50%, we decided to refine this structure in the non-centrosymmetric space group $Pca2_1$.

4: A part of the main molecule is refined with a disorder model comprising two positions.

5: Three disordered molecules of THF were refined over two positions each. The respective atoms were refined isotropically.

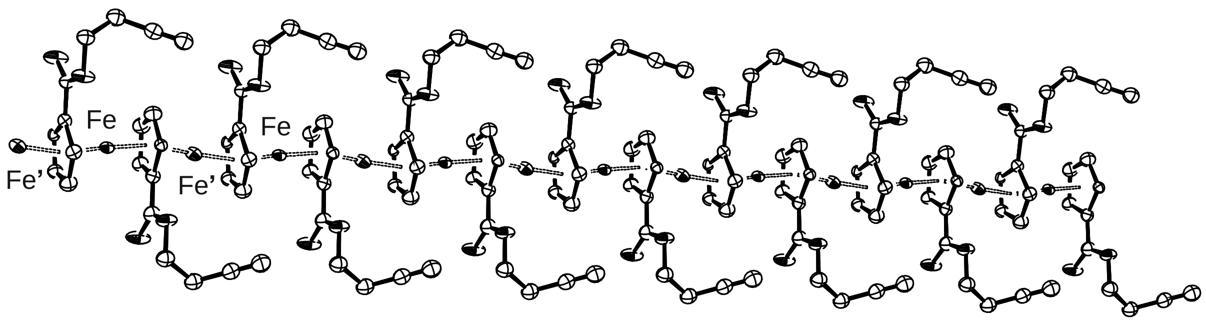

Figure S15. ORTEP diagram of **1a** with thermal displacement drawn at 50% probability; displayed is the chain like structure of the ferrocene units connected via Fe' in a disordered position occupied by 26%; hydrogen atoms are omitted for clarity.

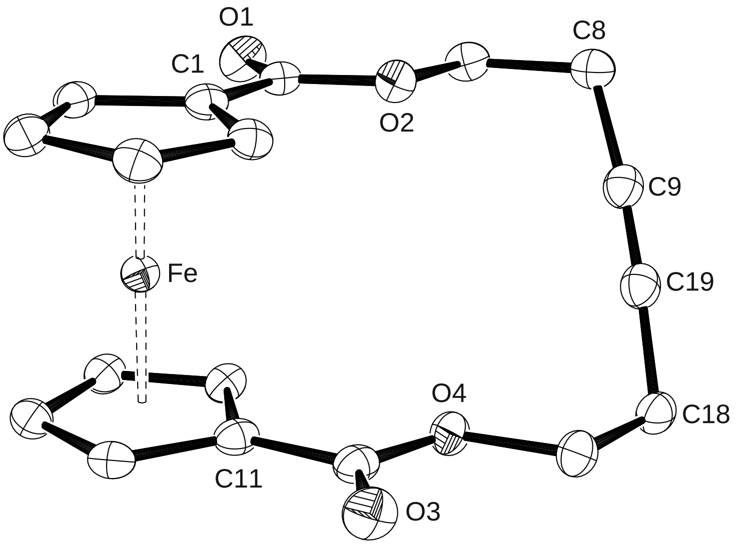

Figure S16. ORTEP diagram of the triclinic polymorph **2a**[a] obtained from the crystallisation of **2a** from THF with Et_2O with thermal displacement drawn at 50% probability; hydrogen atoms are omitted for clarity. Selected bond lengths [Å] and angles [°]: Fe–Ct1 1.6559(3), Fe–Ct11 1.6574(3), Ct1–Fe–Ct11 179.35(2), C9–C19 1.193(3), C8–C9–C19 175.5(2), C9–C19–C18 177.1(2).

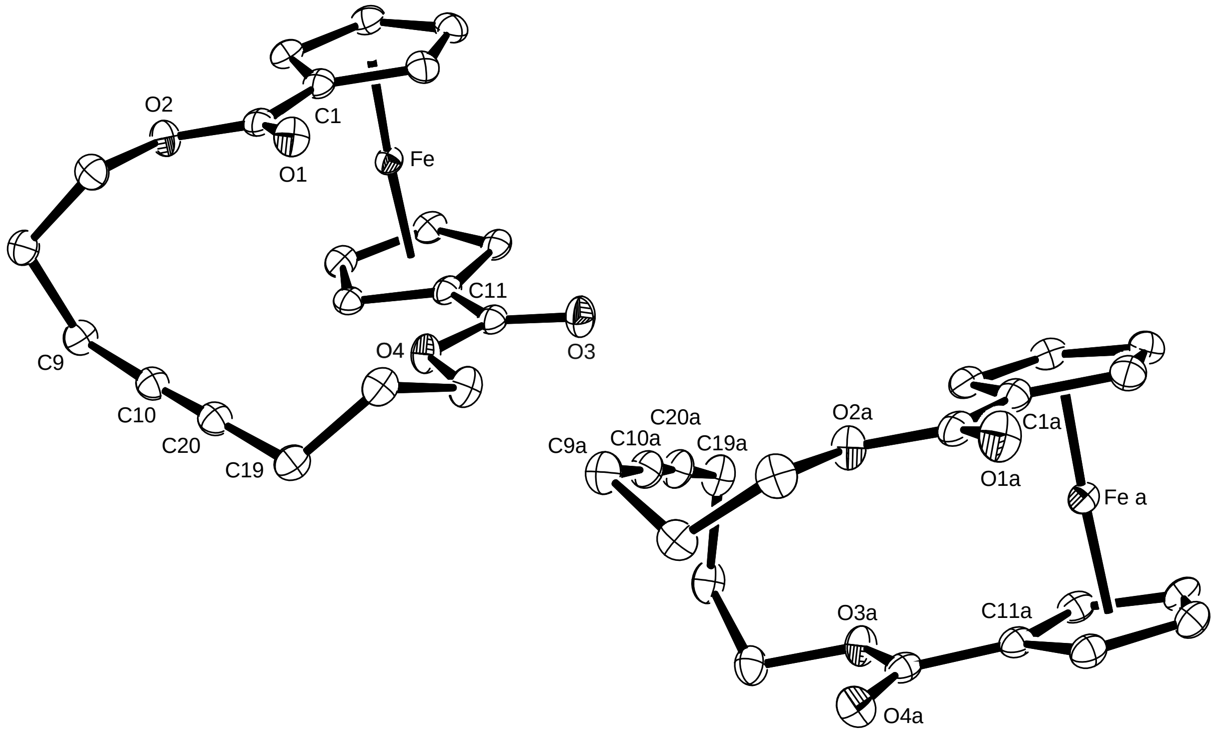

Figure S17. ORTEP diagram of **2b** with thermal displacement drawn at 50% probability; both molecules of the asymmetric unit are shown, hydrogen atoms are omitted for clarity. Selected bond lengths [Å] and angles [°]: Fe–Ct1 1.6558(2), Fe–Ct11 1.6548(2), Ct1–Fe–Ct11 176.83(2), C10–C20 1.198(3), C9–C10–C20 177.6(2), C10–C20–C19 179.1(2); Fe a–Ct1a 1.6541(3), Fe a–Ct11a 1.6534(3), Ct1a–Fe a–Ct11a 177.85(2), C10a–C20a 1.197(3), C9a–C10a–C20a 177.9(2), C10a–C20a–C19a 176.3(2).

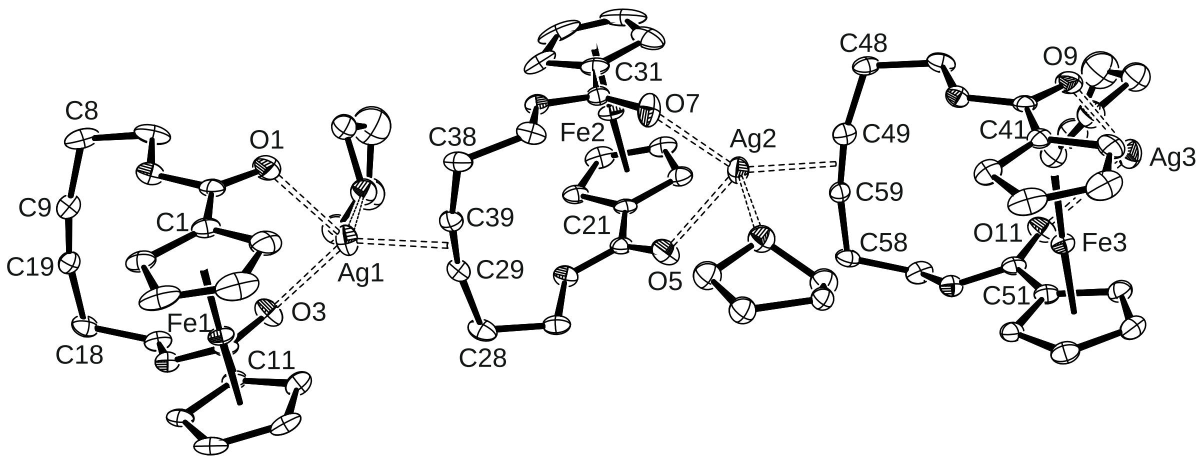

Figure S18. ORTEP diagram of **5** with thermal displacement drawn at 50% probability; hydrogen atoms are omitted for clarity, only the main positions of the disorderes thf molecules are given. Selected bond lengths [Å] and angles [°] are summarized in Table S1 and Table S2.

Table S1. Selected bond lengths [Å] and angles [°] of **5**; bonds with metal participation.

	Ct	Ct‘	Ct–Fe–Ct‘		O	O‘	O(thf)
Fe1	1.6814(6)	1.6524(6)	168.20(4)	Ag1	2.357(3)	2.284(3)	2.430(5)
Fe2	1.6539(6)	1.6480(6)	174.49(4)	Ag2	2.342(3)	2.287(3)	2.451(5)
Fe3	1.6538(6)	1.6552(6)	175.16(4)	Ag3	2.302(3)	2.334(3)	2.427(3)

Table S2. Selected bond lengths [Å] and angles [°] of **5**; bonds with alkyne participation.

		C–C≡C	C≡C–C	Ag–(C≡C)
C9–C19	1.217(6)	168.9(5)	167.7(5)	2.2453(3)
C29–C39	1.209(6)	166.6(5)	167.2(5)	2.2321(3)
C49–C59	1.230(6)	166.3(5)	167.8(4)	2.2390(3)

Table S3. Selected crystallographic data for compounds **1a**, **1b** and the orthorhombic polymorph of **2a** measured by X-ray structure analysis.

	**1a**	**1b**	**2a°**
CCDC	1870273	1870274	1870275
Empirical formula	$C_{20}H_{18}FeO_4$	$C_{22}H_{22}FeO_4$	$C_{18}H_{16}FeO_4$
M [g mol^{-1}]	378.19	406.25	352.16
T [K]	100(2)	100(2)	100(2)
λ [Å]	1.54184	1.54184	0.71073
Crystal system	orthorhombic	monoclinic	orthorhombic
Space group	P c a 2_1	*P*2_1/*c*	*Pbcn*
a [Å]	11.2911(4)	11.5038(4)	7.7798(3)
b [Å]	6.7989(3)	10.3533(3)	20.0621(6)
c [Å]	21.8070(8)	7.9683(3)	9.4277(3)
α [°]	90	90	90
β [°]	90	92.284(3)	90
γ [°]	90	90	90
V [Å^3]	1674.06(11)	948.29(6)	1471.47(9)
Z	4	2	4
Density [g cm^{-3}]	1.501	1.423	1.590
m [mm^{-1}]	7.413	6.582	1.043
F(000)	781	424	728
Crystal size [mm]	0.23x0.08x0.27	0.18x0.16x0.08	0.28x0.10x0.07
Theta range [°]	4.05 - 76.37	3.85 - 76.25	0.28 - 29.13
Reflexions collected	25065	9891	27708
Independent reflections	3494	1968	1990
R(int)	0.7390	0.0302	0.0374
Data/restraints/parameters	3494/121/237	1968/0/124	1990/0/105
GooF	1.103	1.087	1.063
R1 (F, >4σ(F))	0.0808	0.0285	0.0289
wR2 (F2, all data)	0.1980	0.0770	0.0714
max. Δρ [e Å^{-3}]	1.080/−0.656	0.204/−0.407	0.441/−0.323

Table S4. Selected crystallographic data for the triclinic polymorph of **2a** and compounds **2b** and **4** measured by X-ray structure analysis.

	**2a**[a]	**2b**	**4**
CCDC	1870276	1870277	1870278
Empirical formula	$C_{18}H_{16}FeO_4$	$C_{20}H_{20}FeO_4$	$C_{18}H_{16}F_6FeO_4Sb$
M [g mol^{-1}]	352.16	380.21	587.91
T [K]	100(2)	100(2)	100(2)
λ [Å]	1.54184	1.54184	0.71073
Crystal system	triclinic	triclinic	monoclinic
Space group	$P\bar{1}$	$P\bar{1}$	$P\,2_1/c$
a [Å]	9.6657(7)	6.7796(3)	7.7742(8)
b [Å]	9.9367(9)	16.0367(6)	20.746(2)
c [Å]	10.1480(8)	16.3279(7)	12.4205(14)
α [°]	64.259(8)	110.086(4)	90
β [°]	64.848(7)	94.337(3)	96.901(4)
γ [°]	62.995(8)	90.276(3)	90
V [Å^3]	750.04(11)	1661.56(13)	1988.7(4)
Z	2	4	4
Density [g cm^{-3}]	1.599	1.520	1.964
m [mm^{-1}]	8.225	7.469	2.165
F(000)	364	792	1148
Crystal size [mm]	0.15x0.12x0.11	0.303x0.112x0.075	0.20x0.08x0.03
Theta range [°]	5.05 - 76.26	3.336 to 76.220	1.921 - 27.530
Reflexions collected	14143	67456	81709
Independent reflections	3122	6937	4570
R(int)	0.0317	0.0570	0.0550
Data/restraints/parameters	3122/0/208	6937/0/451	4570/0/31
GooF	1.074	1.066	1.109
R1 (F, >4σ(F))	0.0304	0.0313	0.0266
wR2 (F2, all data)	0.0863	0.0841	0.0518
max. Δρ [e Å^{-3}]	0.465/−0.524	0.522/−0.607	0.575/−0.845

Table S5. Selected crystallographic data for coordination polymer **5** measured by X-ray structure analysis.

	**5**
CCDC	1870279
Empirical formula	$C_{66}H_{72}Ag_3F_{18}Fe_3O_{15}Sb_3$
M [g mol^{-1}]	2303.65
T [K]	100(2)
λ [Å]	1.54184
Crystal system	monoclinic
Space group	$P\ 2_1/c$
a [Å]	14.99930(14)
b [Å]	13.44550(10)
c [Å]	37.6178(3)
α [°]	90
β [°]	91.8395(8)
γ [°]	90
V [Å^3]	7582.59(11)
Z	4
Density [g cm^{-3}]	2.018
M [mm^{-1}]	19.814
F(000)	4488
Crystal size [mm]	0.20x0.19x0.05
Theta range [°]	3.49 - 76.33
Reflexions collected	215741
Independent reflections	15843
R(int)	0.0843
Data/restraints/parameters	15843/30/961
GooF	1.115
R1 (F, >4σ(F))	0.0387
wR2 (F2, all data)	0.0978
max. Δρ [e Å^{-3}]	1.200/−1.440

5. References

1. Haberlag, B.; Freytag, M.; Daniliuc, C. G.; Jones, P. G.; Tamm, M. *Angew. Chem. Int. Ed.* **2012**, *51*, 13019–13022.
2. Petrov, A. R.; Jess, K.; Freytag, M.; Jones, P. G.; Tamm, M. *Organometallics* **2013**, *32*, 5946–5954.
3. Curran, T. P.; Lawrence, A. P.; Murtaugh, T. S.; Ji, W.; Pokharel, N.; Gober, C. B.; Suitor, J. *J. Organomet. Chem.* **2017**, *846*, 24–32.
4. CrysalisPRO Software System; Rigaku Oxford Diffraction: Rigaku Corporation, Oxford, UK.
5. SAINT; Bruker: Bruker AXS Inc., Madison, Wisconsin, USA.
6. Sheldrick, G. M. *Acta crystallographica. Section C, Structural chemistry* **2015**, *71*, 3–8.
7. Sheldrick, G. M. *Acta crystallographica. Section A, Foundations and advances* **2015**, *71*, 3–8.
8. Dolomanov, O. V.; Bourhis, L. J.; Gildea, R. J.; Howard, J. A. K.; Puschmann, H. *J Appl Crystallogr* **2009**, *42*, 339–341.
